# Development of a metabolite calculator for diagnosis of pancreatic cancer

**DOI:** 10.1002/cam4.6233

**Published:** 2023-06-23

**Authors:** Munseok Choi, Minsu Park, Sung Hwan Lee, Min Jung Lee, Young‐Ki Paik, Sung Il Jang, Dong Ki Lee, Sang‐Guk Lee, Chang Moo Kang

**Affiliations:** ^1^ Department of Surgery, Yongin Severance Hospital Yonsei University College of Medicine Yongin‐si South Korea; ^2^ Department of Information and Statistics Chungnam National University Daejeon South Korea; ^3^ Department of Surgery, CHA Bundang Medical Center CHA University South Korea; ^4^ Yonsei Proteome Research Center and Department of Integrated OMICS for Biomedical Science and Department of Biochemistry, College of Life Science and Biotechnology Yonsei University Seoul South Korea; ^5^ Department of Internal Medicine, Gangnam Severance Hospital Yonsei University College of Medicine Seoul South Korea; ^6^ Department of Laboratory Medicine, Severance Hospital Yonsei University College of Medicine Seoul South Korea; ^7^ Department of Surgery, Severance Hospital Yonsei University College of Medicine Seoul South Korea

**Keywords:** biomarker, calculator, diagnosis, metabolomics, pancreatic cancer

## Abstract

**Background:**

Carbohydrate antigen (CA) 19–9 is a known pancreatic cancer (PC) biomarker, but is not commonly used for general screening due to its low sensitivity and specificity. This study aimed to develop a serum metabolites‐based diagnostic calculator for detecting PC with high accuracy.

**Methods:**

A targeted quantitative approach of direct flow injection‐tandem mass spectrometry combined with liquid chromatography–tandem mass spectrometry was employed for metabolomic analysis of serum samples using an Absolute IDQ™ p180 kit. Integrated metabolomic analysis was performed on 241 pooled or individual serum samples collected from healthy donors and patients from nine disease groups, including chronic pancreatitis, PC, other cancers, and benign diseases. Orthogonal partial least squares discriminant analysis (OPLS‐DA) based on characteristics of 116 serum metabolites distinguished patients with PC from those with other diseases. Sparse partial least squares discriminant analysis (SPLS‐DA) was also performed, incorporating simultaneous dimension reduction and variable selection. Predictive performance between discrimination models was compared using a 2‐by‐2 contingency table of predicted probabilities obtained from the models and actual diagnoses.

**Results:**

Predictive values obtained through OPLS‐DA for accuracy, sensitivity, specificity, balanced accuracy, and area under the receiver operating characteristic curve (AUC) were 0.9825, 0.9916, 0.9870, 0.9866, and 0.9870, respectively. The number of metabolite candidates was narrowed to 76 for SPLS‐DA. The SPLS‐DA‐obtained predictive values for accuracy, sensitivity, specificity, balanced accuracy, and AUC were 0.9773, 0.9649, 0.9832, 0.9741, and 0.9741, respectively.

**Conclusions:**

We successfully developed a 76 metabolome‐based diagnostic panel for detecting PC that demonstrated high diagnostic performance in differentiating PC from other diseases.

## 1 INTRODUCTION

Pancreatic cancer (PC) is one of the most fatal malignant diseases of the gastrointestinal tract.^1^ Margin‐negative resection is essential for ensuring long‐term survival.^2^ However, less than 20% of patients with PC are resectable, and most patients are found to have advanced or metastatic disease at the initial stage of diagnosis, resulting in an overall 5‐year survival rate of less than 10%.^3^ No improvement in survival has been noted in patients with PC during the past few decades, with PC likely to become the second most deadly form of human cancer by 2030.^4^

However, advances in surgical approaches, surgical pancreatectomy techniques, perioperative management, and potent chemotherapeutic agents are expected to improve the long‐term survival of patients with PC^5^, ^6^, ^7^, ^8^ as long as more resectable PC could be found in real clinical practice. Therefore, many investigators have attempted to identify potential biomarkers for the early detection of PC; however, due to their unsatisfactory diagnostic performance within the general population, there are currently no clinically relevant biomarkers for the accurate diagnosis of PC.^9^

The integration of metabolomics in cancer biomarker research, including that for PC, is an emerging field.^10^, ^11^ Metabolomics is the latest type of multi‐omics approaches that have included genomics, transcriptomics, and proteomics, and focus on phenotypic characteristics rather than genetic profiles.^12^ Metabolomics methodology aims to identify and estimate the relative changes in abundance of endogenous metabolites during conditions of health and disease, which can then be used to support the identification of biomarkers and potential targets and the development of new therapeutics for the diagnosis and treatment of PC.^13^ Furthermore, the metabolomic characteristics of patients with PC may reflect the functional aspects of PC as a whole.^14^

Based on a targeted metabolomic approach using high performance liquid chromatography (HPLC), we previously investigated the potential role of serum metabolites for predicting the survival of patients with resected PC. We found that among 157 serum metabolites detected preoperatively in patients with resected PC, serum carbohydrate antigen (CA) 19–9 and three phosphatidyl choline derivatives (PC.aa.C38_4, PC.ae.C42_5, and PC.ae.C38_6) can be used preoperatively to estimate 1‐year disease‐free survival.^15^ This provides a preoperative risk estimation and additional information useful during the decision‐making process regarding surgical resection.

In the current study, we aimed to develop a highly accurate diagnostic serum metabolomic panel for detection of PC with high accuracy. Such a preoperative serum metabolite‐based diagnostic tool could provide all‐in‐one clinical advantages to both the detection of PC and in predicting early recurrence of resected PC. This would support the potential of providing tailored treatment approaches for patients with resectable PC.

## 2 MATERIALS AND METHODS

### 2.1 Study population

**FIGURE 1:**
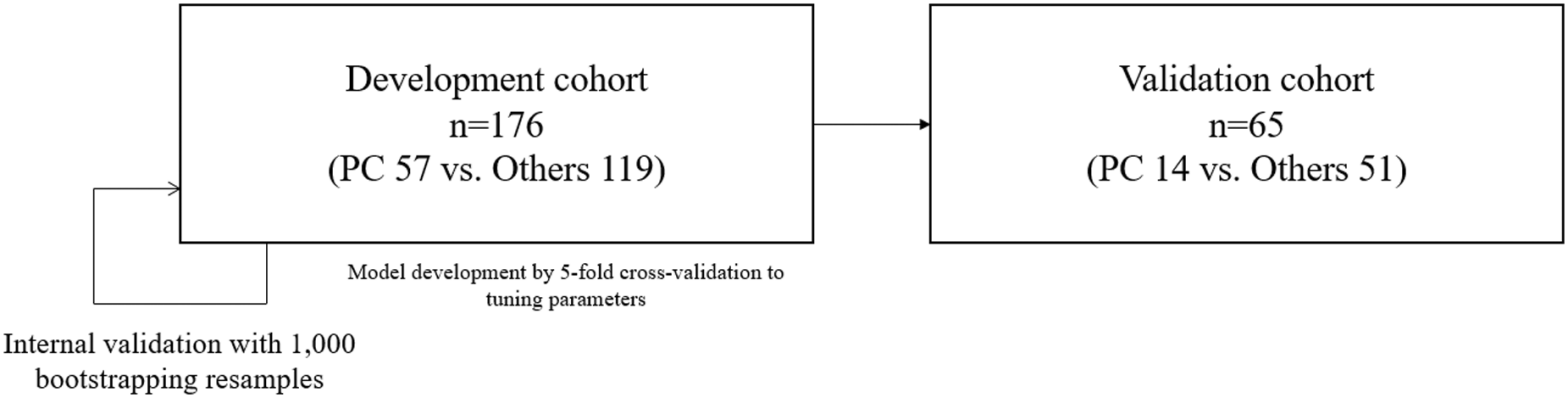
Flow diagram of the study cohorts.

Two separate clinical enrollments were used in the study to obtain blood samples for metabolomic analysis. The first enrollment was the source of blood samples from a development cohort of 186 individuals that were collected by the Biobank, Severance Hospital, Seoul, South Korea. Among the first enrollment participants, ten patients with PC had missing values and were excluded from the study. Thus, the development cohort used for model building included blood samples from 176 individuals of which 57 were patients with PC and 119 were patients with other diseases (Figure 1). The second cohort was used as the validation cohort and it consisted of 65 individuals who provided bioresources to the National Biobank of Korea, The Center for Disease Control and Prevention, Republic of Korea. The validation cohort included 14 patients with PC and 51 patients that had other diseases.

### 2.2 Sample preparation

The blood samples were collected after a fasting period of at least 8 h prior to sampling. The serum separating tube were centrifuged to separate the serum from the blood clot (4°C, 10 min, 2500 × g) within 40 min after sample collection. Then 1 mL serum was aliquoted into the pre‐cooled and labeled storage vials. The serum aliquots were frozen immediately and stored below −80 °C until use.

### 2.3 Detection of preoperative serum metabolites

A targeted quantitative approach was used to analyze the serum samples for metabolomic analysis. Specifically, direct flow injection‐tandem mass spectrometry (FIA‐MS/MS) combined with liquid chromatography–tandem mass spectrometry (LC–MS/MS) was performed using an Absolute IDQ™ p180 Kit (BIOCRATES Life Sciences AG, Innsbruck, Austria) as described previously.^15^ The kit was able to simultaneously quantify 188 metabolites, including 21 amino acids, 21 biogenic amines, 40 acylcarnitines, 90 glycerophospholipids (14 lysophosphatidylcholines and 76 phosphatidylcholines [PC.Aa.Cx:y or PC.ae.Cx:y]), 15 sphingolipids, and 1 hexose. Cx:y denotes the lipid side chain configuration, where x indicates the number of carbons in the side chain and y indicates the number of unsaturated chains. Among these 188 metabolites, 21 amino acids and 21 biogenic amines were analyzed by LC–MS/MS while the other 146 metabolites were analyzed by FIA‐MS/MS. The serum samples were processed in strict accordance with the manufacturer's instructions. Briefly, 10 μL of the supplied internal standard solution was added to each well of a 96‐well extraction plate, followed by 10 μL of each serum sample being added to the appropriate wells. The plate was dried under a gentle stream of nitrogen. The samples were derivatized with phenylisothiocyanate and eluted with 5 mM ammonium acetate in methanol. The samples were diluted with 40% methanol in water (15:1) for LC–MS/MS analysis or a proprietary running solvent provided by BIOCRATES Life Sciences AG (20:1) for FIA‐MS/MS. The samples were analyzed in 96‐well plates using a QTRAP 5500 mass spectrometer (SCIEX, Woodlands Central, Singapore) coupled with an Agilent 1290 series HPLC system. For LC–MS/MS analysis in positive mode, 5 μL of the sample extract were injected onto an Agilent Zorbax Eclipse XDB C18, 3.0 × 100 mm, 3.5 μm protected by an SecurityGuard pre‐column C18, 4 × 3 mm (Phenomenex) at 50°C using a 9.5 min solvent gradient employing 0.2% formic acid in water (mobile phase A) and 0.2% formic acid in acetonitrile (mobile phase B). LC–MS/MS data were imported into the SCIEX application Analyst™ for peak integration, calibration, and concentration calculations. Twenty microliters of the sample extract were used in the FIA‐MS/MS in positive mode to measure acylcarnitines, glycerophospholipids, and sphingolipids, while hexoses were monitored in a subsequent run in negative mode. All FIA injections were carried out using the mobile phase prepared by Biocrates Solvent I in isocratic mode. The LC and MS settings for LC–MS/MS and FIA‐MS/MS mode are described in Tables S5 and S6. Analytical performance was monitored by three levels of quality control (QC) samples (low, middle, high concentration). Three levels of QC samples were placed at the beginning of analytical run. For the QC sample with middle concentration (QC2), additional control samples were placed at the middle and end of analytical run. The LC–MS/MS data from Analyst™ and FIA‐MS/MS data were analyzed using MetIDQ™ software (BIOCRATES Life Sciences AG). To correct for batch effect for both FIA‐MS/MS and LC–MS/MS data, we normalized the data using QC2 results of each batch. The following conditions were set as data quality requirements for each metabolite: (1) the coefficient of variance for the metabolites in the quality control samples was <25%; and (2) 100% of the measured metabolite concentrations in the subject samples was greater than the limit of detection. A total of 72 of the 188 metabolites were not detected in one or more samples and were therefore excluded from the subsequent analysis (Table S4). The remaining 116 metabolites were selected for statistical analysis.

### 2.4 Model development and statistical analysis

Linear discriminant analysis was used for classification, and a five‐fold cross‐validation method was used to select the tuning parameters of the model. This approach was used to separate the datasets into training and test datasets, to prevent overestimation, and estimate some parameters of the model. Discriminant models were developed and internally validated using the development cohort. These models were then applied to the validation cohort for external validation.

The types of diseases of the patients and the corresponding number of patients are shown in Table 1. In the development cohort, 57 patients had PC. The remaining 119 individuals were normal cases or had other types of diseases, including gallbladder stones, chronic pancreatitis, pancreatic neuroendocrine tumors, thyroid papillary carcinomas, breast cancer, lung cancer, or hepatocellular carcinomas. Meanwhile, the validation cohort consisted of disease groups that were similar to those in the development cohort (Table 1).

**TABLE 1:** Development and validation dataset construction.

Disease	Development cohort (*n* = 176)	Validation cohort (*n* = 65)
Pancreatic ductal adenocarcinoma	57	14
Normal	27	26
Gallbladder stone	20	7
Chronic pancreatitis	24	12
Pancreatic neuroendocrine tumor	9	2
Thyroid papillary carcinoma	10	–
Breast cancer	10	–
Lung cancer	10	–
Hepatocellular carcinoma	9	2
Stomach cancer	–	2

Continuous variables are presented as median values with interquartile ranges (IQR) and were compared using the Mann–Whitney U‐test. Because serum metabolites are highly correlated with each other, orthogonal partial least squares discriminant analysis (OPLS‐DA) was used to distinguish patients with PC from those with other diseases based on characteristics of the serum metabolites.^16^ Variable importance in the projection (VIP) ranks indicated the overall contribution of each metabolite to the OPLS‐DA model. The variables with a VIP >1.0, and Spearman's correlation coefficient of |r| > 0.265 were considered to be associated with discrimination for patients with PC. The *p*‐value of the correlation coefficient was adjusted using Bonferroni's correction. Default 7‐cross validation and response permutation testing with 200 permutations were conducted to evaluate the quality and validity of the OPLS‐DA model. Moreover, the OPLS‐DA models were assessed for predictability through p‐values obtained from analysis of variance of cross‐validated residuals (CV‐ANOVA) implemented by SIMCA (version 17.0)^17^ Metabolites responsible for metabolic differences between two groups were visually represented in corresponding loading and score plots. We also performed sparse partial least squares discriminant analysis (SPLS‐DA), which incorporated simultaneous dimension reduction and variable selection, and the OPLS‐DA method using all serum metabolites.^18^ A classification algorithm developed by Chung and Keles was used to perform SPLS‐DA, which is advantageous in terms of utilization as it can be predictive in the diagnosis of cancers using only select significant serum metabolites, not all variables.^18^ In the current study, five‐fold cross‐validation was used to determine the optimal threshing parameter and the optimal number of hidden components. To analyze the constructed models using a net benefit approach, decision curve analysis was implemented.^19^

Performance of the predicted probability obtained from the model was compared with the actual diagnoses using two‐by‐two contingency tables to evaluate sensitivity, specificity, accuracy, balanced accuracy, and the area under the receiver operating characteristic curve (AUC) for the development cohort and external validation cohort datasets. In addition, a 1000 bootstrap procedure was employed for internal validation. Significance was confirmed according to whether the 95% confidence interval for the differences between the two measurements included zero. Statistical significance was set at *p* < 0.05. All statistical analyses were performed using the R package, version 4.1.1 (R Foundation for Statistical Computing, Vienna, Austria). All patients provided written informed consent prior to surgery, and the study was approved by the Institutional Review Board of Yonsei University College of Medicine (registration date: June 18, 2019; registration number: 4–2019‐0415).

## 3 RESULTS

### 3.1 Discrimination of PC cases from controls using the OPLS‐DA model

**FIGURE 2:**
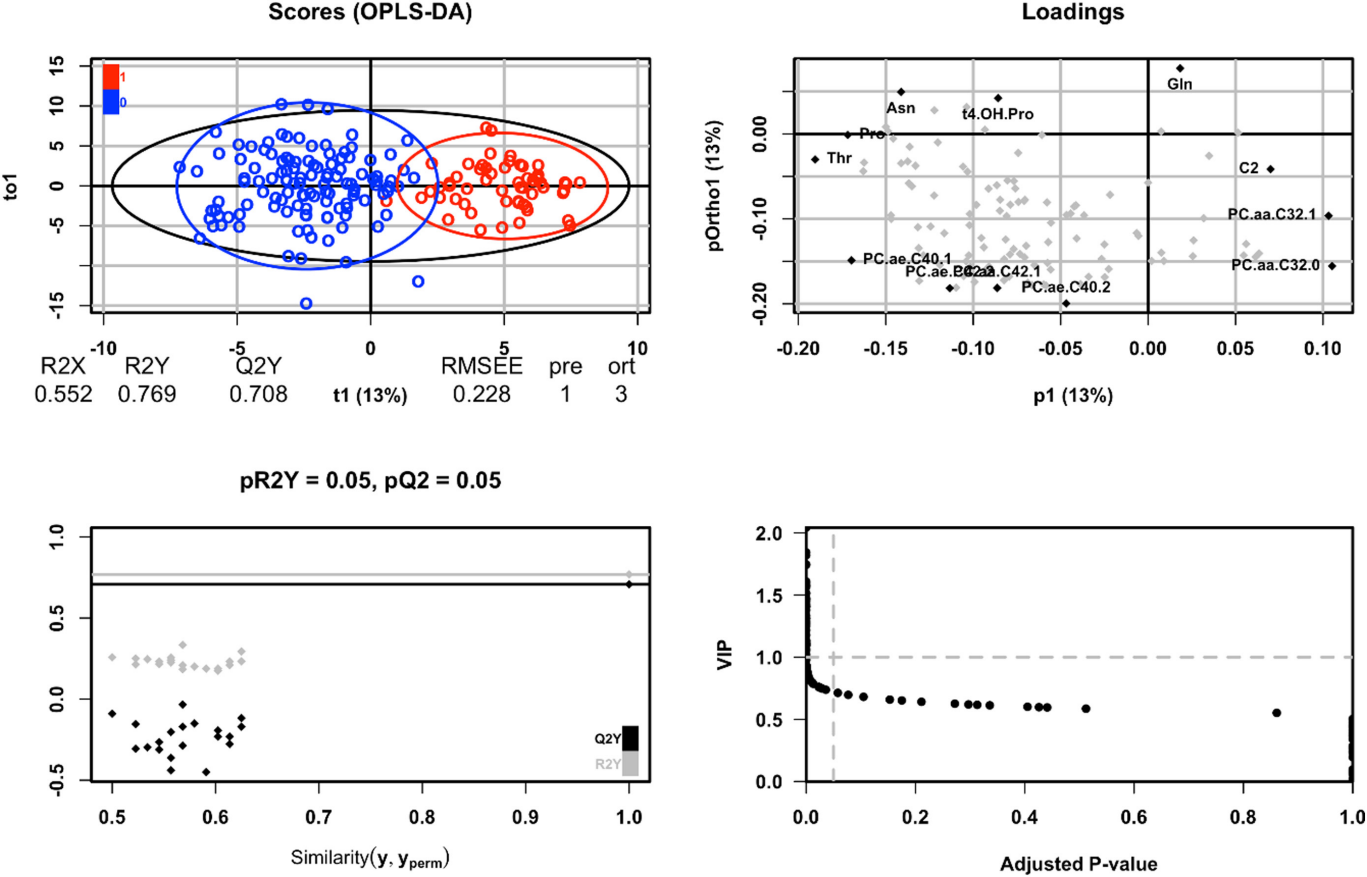
Plots of the OPLS‐DA model. (Top left) OPLS‐DA score plot of serum metabolic profiles on samples from pancreatic cancer patients and others. (Top right) Loading plot of the OPLS‐DA model. Metabolites with the most extreme values for each loading are shown in black and labeled. (Bottom left) Permutation test plot on the OPLS‐DA model. Seven‐cross‐validation and two hundred permutations were performed, and the results of R^2^ and Q^2^ values were dotted. (Bottom right) Relationship between VIP values obtained from the OPLS‐DA model and adjusted p‐values from Pearson correlation test. OPLS‐DA, orthogonal partial least squares‐discriminant analysis; VIP, variable importance in the projection.

Serum samples from 176 individuals in the development cohort set were evaluated. Of the 188 metabolites measured, 72 were excluded based on the data quality criteria. As a result, a total of 116 endogenous metabolites were included in the statistical analysis. A representative OPLS‐DA scatterplot of this classification model based on the development set is shown in Figure 2. Very little overlap between the cases and controls was observed. The permutation test statistically assessed class separation of the metabolites by measuring and visually illustrating the statistical significance of case and control metabolome separations. There was a lack of overfitting among patients with PC vs. those without PC in the development set. The performance indices based on 200 permutations were R^2^Y = 0.769 and Q^2^Y = 0.708, and the p‐value for CV‐ANOVA was <0.001. SPLS‐DA was then performed to highlight the metabolites that best discriminated cases of patients with PC from the controls. Seventy‐six metabolites with coefficients in SPLS‐DA are listed in Table 2.

**TABLE 2:** List of 76 selected metabolites and coefficients in the SPLS‐DA.

Metabolite	Coefficient	Metabolite	Coefficient	Metabolite	Coefficient
Alanine	−0.91	lysoPhosphatidylcholine acyl C18:1	0.37	Phosphatidylcholine acyl‐alkyl C36:4	−0.08
Asparagine	−2.13	lysoPhosphatidylcholine acyl C18:2	−1.43	Phosphatidylcholine acyl‐alkyl C38:0	0.07
Aspartate	−0.27	lysoPhosphatidylcholine acyl C20:3	−0.04	Phosphatidylcholine acyl‐alkyl C38:1	−0.79
Citrulline	−1.96	lysoPhosphatidylcholine acyl C20:4	−0.71	Phosphatidylcholine acyl‐alkyl C38:2	−0.83
Glutamate	−0.24	Phosphatidylcholine diacyl C32:0	4.28	Phosphatidylcholine acyl‐alkyl C38:3	−0.16
Glycine	−1.63	Phosphatidylcholine diacyl C32:1	3.11	Phosphatidylcholine acyl‐alkyl C38:4	0.18
Histidine	−1.67	Phosphatidylcholine diacyl C32:2	−0.99	Phosphatidylcholine acyl‐alkyl C40:1	−1.86
Isoleucine	−1.32	Phosphatidylcholine diacyl C32:3	0.71	Phosphatidylcholine acyl‐alkyl C40:3	−0.23
Leucine	−1.91	Phosphatidylcholine diacyl C34:2	0.66	Phosphatidylcholine acyl‐alkyl C40:4	−0.37
Lysine	−0.93	Phosphatidylcholine diacyl C34:4	−0.20	Phosphatidylcholine acyl‐alkyl C40:5	0.47
Ornithine	−1.56	Phosphatidylcholine diacyl C36:2	0.05	Phosphatidylcholine acyl‐alkyl C42:1	0.05
Phenylalanine	−0.97	Phosphatidylcholine diacyl C36:3	0.74	Phosphatidylcholine acyl‐alkyl C42:2	−0.77
Proline	−2.63	Phosphatidylcholine diacyl C36:6	−0.10	Phosphatidylcholine acyl‐alkyl C42:3	−0.66
Serine	−1.29	Phosphatidylcholine diacyl C38:1	−2.02	Phosphatidylcholine acyl‐alkyl C44:3	−0.82
Threonine	−3.16	Phosphatidylcholine diacyl C40:2	−0.45	Phosphatidylcholine acyl‐alkyl C44:4	−0.83
Tryptophan	−1.17	Phosphatidylcholine diacyl C40:3	−0.16	Phosphatidylcholine acyl‐alkyl C44:5	−0.20
Tyrosine	−0.96	Phosphatidylcholine diacyl C42:1	0.42	Phosphatidylcholine acyl‐alkyl C44:6	0.17
Valine	−1.85	Phosphatidylcholine diacyl C42:2	−0.38	Hydroxysphingomyelin C22:1	−1.54
Creatinine	−1.12	Phosphatidylcholine diacyl C42:4	0.13	Hydroxysphingomyelin C22:2	−1.13
trans‐4‐Hydroxyproline	−0.87	Phosphatidylcholine diacyl C42:5	−0.18	Hydroxysphingomyelin C24:1	−1.05
Taurine	−0.85	Phosphatidylcholine acyl‐alkyl C32:2	0.37	Sphingomyelin C16:1	0.01
Acetyl‐L‐carnitine	2.27	Phosphatidylcholine acyl‐alkyl C34:2	0.21	Sphingomyelin C18:1	0.65
lysoPhosphatidylcholine acyl C16:0	−0.09	Phosphatidylcholine acyl‐alkyl C34:3	0.07	Sphingomyelin C24:0	−0.56
lysoPhosphatidylcholine acyl C16:1	0.50	Phosphatidylcholine acyl‐alkyl C36:1	−0.02	Sphingomyelin C26:1	2.81
lysoPhosphatidylcholine acyl C17:0	−0.28	Phosphatidylcholine acyl‐alkyl C36:2	−0.28		
lysoPhosphatidylcholine acyl C18:0	−0.01	Phosphatidylcholine acyl‐alkyl C36:3	−0.29		

Abbreviation: SPLS‐DA, sparse partial least squares discriminant analysis.

### 3.2 Diagnostic performance of the OPLS‐DA and SPLS‐DA models using the development set

OPLS‐DA was performed to identify metabolites responsible for the discrimination between patients with PC and those with other diseases. As shown in Figure 2, the OPLS‐DA score plot showed a clustering tendency between the two groups, indicating obvious metabolic differences. Similar results were found in the heat map as well, indicating that the metabolomic expression of each group was different (Figure S1).

According to the predictive variation between the metabolites and data subjects, 55.2% (R^2^X cum) of the total explained variation in the dataset accounted for 76.9% of the variance in group separation (R^2^Y cum). The cross‐validated predictability of the discrimination analysis model was 70.8% (Q^2^Y cum). Based on the positive R^2^Y and negative Q^2^Y values of the permutation test, the performance of the OPLS‐DA model was satisfactory. In addition, the OPLS‐DA loading plot in Figure 2 shows the distribution of discriminating metabolites, with metabolites far from the origin in each direction being indicated as black points. Finally, the adjusted *p*‐values for the Spearman's correlation coefficients and VIP values were evaluated (Figure 2). Significant metabolites satisfying the adjusted *p*‐value <0.05 and VIP value >1.0 were identified. The results are summarized in Tables S1 and S2.

After establishing the OPLS‐DA and SPLS‐DA classification models using the development cohort, the discrimination performance of the models were confirmed. The number of hidden components and threshold parameter for the SPLS‐DA model were estimated as 3 and 0.7, respectively. As a result, only 76 metabolites were included in the SPLS‐DA model, while all 116 metabolites were used in the OPLS‐DA model. The performance of the OPLS‐DA and SPLS‐DA classification models are summarized in Table 3. OPLS‐DA identified 56 true positives, one false positive, and one false negative for a sensitivity of 0.9825, specificity of 0.9916, and accuracy of 0.9886. Meanwhile, SPLS‐DA identified 55 true positives, two false positives, and two false negatives for a sensitivity of 0.9773, specificity of 0.9649, and accuracy of 0.9773. Similarly, the balanced accuracy, defined as the average of sensitivity and specificity, was 0.9870 for OPLS‐DA, which was slightly larger than the balanced accuracy balanced accuracy of 0.9741 for SPLS‐DA. The AUC values for OPLS‐DA and SPLS‐DA were 0.9870 and 0.9741, respectively. Overall, the OPLS‐DA model using all 116 metabolites yielded better results for the development cohort compared with that of the SPLS‐DA model. However, the SPLS‐DA model, for which 40 metabolites were excluded, also demonstrated good classifying ability.

**TABLE 3:** Performance of classifiers for metabolites from the development cohort (*n* = 176).

	OPLS‐DA with 116 metabolomes	SPLS‐DA with 76 metabolomes
Accuracy	0.9886	0.9773
Sensitivity	0.9825	0.9649
Specificity	0.9916	0.9832
Balanced accuracy	0.9870	0.9741
AUC	0.9870	0.9741

Abbreviations: AUC, area under the receiver operating characteristic curve; OPLS‐DA, orthogonal partial least squares discriminant analysis; SPLS‐DA, sparse partial least squares discriminant.

Interval validation was also performed using bootstrapping samples to determine whether similar performance was observed for the OPLS‐DA model compared with that of the SPLS‐DA model, even though only 76 metabolites used for the SPLS‐DA model. The internal validation was performed using 1000 bootstrapping samples, and the 95% confidence intervals differences checked. It was determined that there was no difference between the OPLS‐DA and SPLS‐DA models if the differences in the 95% confidence intervals were zero. As shown in Table 4, the differences in terms of accuracy, sensitivity, specificity, and balanced accuracy between the OPLS‐DA and SPLS‐DA models were 0.012, 0.018, 0.008, and 0.013, respectively. As the differences between the two models were positive values, the performance of the OPLS‐DA model was confirmed to be good. However, because the differences in the 95% confidence between the two models were positive and tend to zero, the differences could not be shown to be statistically significant. The lower boundary of the difference in sensitivity between the two models being 0 was the result of the internal validation dataset not containing many patients with PC. Even the AUC values included confidence intervals of zero, confirming the differences between the two models were not statistically significant. Therefore, the performance of the SPLS‐DA model with only 76 metabolites, was similar to that of the OPLS‐DA model using all 116 serum metabolites.

**TABLE 4:** Performance of classifiers for metabolites from the 1000 bootstrapping internal validation data set (*n* = 176).

	OPLS‐DA with 117 metabolomes	SPLS‐DA with 76 metabolomes	Difference
Accuracy (95% CI)	0.988 (0.972, 1)	0.977 (0.955, 0.994)	0.012 (−0.006, 0.034)
Sensitivity (95% CI)	0.982 (0.941, 1)	0.964 (0.905, 1)	0.018 (0, 0.067)
Specificity (95% CI)	0.992 (0.974, 1)	0.983 (0.958, 1)	0.008 (−0.018, 0.037)
Balanced accuracy (95% CI)	0.987 (0.963, 1)	0.974 (0.944, 0.996)	0.013 (−0.004, 0.039)
AUC (95% CI)	0.987 (0.964, 1)	0.974 (0.944, 0.996)	0.013 (−0.016, 0.045)

Abbreviations: AUC, area under the receiver operating characteristic curve; CI, confidence interval, OPLS‐DA, orthogonal partial least squares discriminant analysis; SPLS‐DA, sparse partial least squares discriminant analysis.

The selected 76 metabolites for the SPLS‐DA model and their coefficients are shown in Table 2. Based on the coefficient values of the standardized metabolites, the SPLS‐DA model had the largest absolute coefficient values for “Phosphatidylcholine diacyl C32:0” (4.28), “Threonine” (−3.16), and “Phosphatidylcholine diacyl C32:1” (3.11).

### 3.3 Diagnostic performance of the OPLS‐DA and SPLS‐DA models using the validation set

**FIGURE 3:**
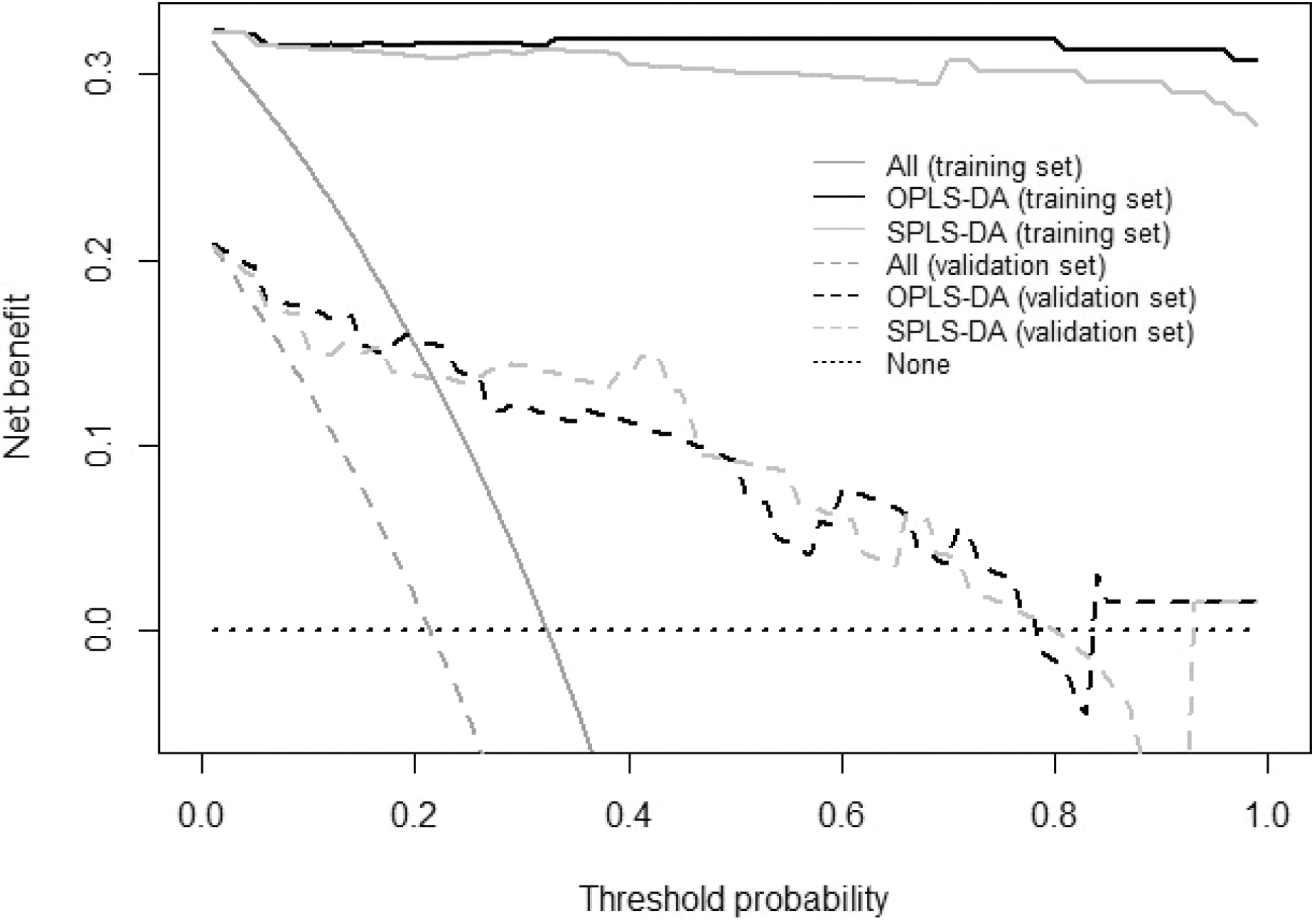
Decision curve analysis. The y‐axis measures the net benefit and the x‐axis indicates the probability threshold to classify a diagnosis of pancreatic cancer. The solid and dashed lines represent the net benefit of the validation and test sets, respectively. The decision curves for the strategies of treating all subjects and treating no subject are expressed as ‘All’ and ‘None’, respectively. The higher curve at any given threshold probability is the optimal discriminant model to maximize net benefit. OPLS‐DA, orthogonal partial least squares‐discriminant analysis.

The performance of the models using the development cohort was evaluated compared with that using the validation cohort. The validation cohort contained 65 patients, including 14 patients that had PC. As shown in Table 5, the specificity of both the OPLS‐DA and SPLS‐DA models was greater than 0.9; however, the sensitivities of the OPLS‐DA and SPLS‐DA models were only 0.7143 and 0.6429, respectively. As a result, the balanced accuracy value was smaller than the accuracy value. The classification ability of the OPLS‐DA and SPLS‐DA models were also evaluated and found to be good with AUC values of approximately 0.8. The fact that there were only 14 patients with PC in the validation cohort resulted in somewhat less accurate results compared with that in the development cohort. However, the overall performance, except for sensitivity, was acceptable. Decision curve analysis graphically showed the clinical usefulness of each model based on the range of threshold probabilities (x‐axis) and the net benefit (y‐axis), the decision curves of the training set (solid line) and validation set (dotted line) were expressed, respectively (Figure 3). The net benefit of the training set was consistently positive in both OPLS‐DA and SPLS‐DA, and the decision curves for both models were similar. Even in the validation set, the decision curves had positive values up to the threshold up to 80%.

**TABLE 5:** Performance of classifiers for metabolites from the external validation cohort (*n* = 65).

Method	OPLS‐DA with 116 metabolomes	SPLS‐DA with 76 metabolomes
Accuracy	0.8769	0.8615
Sensitivity	0.7143	0.6429
Specificity	0.9216	0.9216
Balanced accuracy	0.8179	0.7822
AUC	0.8179	0.7981

Abbreviations: AUC, area under the receiver operating characteristic curve; OPLS‐DA, orthogonal partial least squares discriminant analysis, SPLS‐DA, sparse partial least squares discriminant analysis.

### 3.4 Relevant Metabolites

In the development cohort, 57 of the 176 patients had PC, while the remaining 119 patients had other various cancers and diseases. The baseline characteristics of the study patients in the development cohort are shown in Table S3. Among the 116 metabolites shown in Table S3, there were 95 metabolites that demonstrated statistical significance between the two groups, accounting for approximately 82% of the metabolites. All metabolites starting with ‘lysoPC.a.C' or ‘SM.OH.C' demonstrated significance. In addition, all 76 metabolites selected for the SPLS‐DA model were statistically significant (Table 5).

### 3.5 Applying random sampling to the diagnostic panel

Since the dependent variables used in this study were dichotomous data, not continuous data, the PC diagnosis was predicted by generalizing through the inverse logit function. The metabolites used to construct the SPLS‐DA model were normalized by correcting the average and standard deviation of each metabolite to compare the scales of each metabolite equally. The predicted values were then derived by multiplying the normalized values of the 76 metabolites by each of the specified designated metabolite coefficients shown in Table 2 and by summing the multiplied values with the intercept value of −11.665. Finally, after calculating the predicted PC diagnosis probability by applying the inverse logit function, we determined the correctness of a PC diagnosis.

## 4 DISCUSSION

PC is a unique gastrointestinal cancer that has not improved in terms of oncologic outcomes over the past few decades.^20^ However, advances during the last decade in diagnostic approaches, perioperative management, radiotherapy techniques, and treatment strategies for advanced PC have resulted in modest incremental progress in patient outcomes. With advances in surgical techniques and perioperative management strategies, margin‐negative pancreatectomy is now safely performed at experienced treatment centers.^21^, ^22^ In addition, effective and potent chemotherapeutic agents have been introduced that result in significant improvement in survival differences for patients with PC.^5^, ^23^ However, while margin‐negative resection is currently the most effective monotherapy for treating PC, resection as an option is in less than 20% of the cases at the initial stage of diagnosis. Therefore, the survival outcome of patients with PC could be prominently improved if PC could be more frequently detected clinically at an resectable stage.

Serum CA19‐9 is currently widely used as a biomarker for the detection and evaluation of post‐treatment PC. However, its clinical availability is thought to be limited for monitoring responses to therapy and predicting prognosis. The value of CA 19–9 as a diagnostic marker is highly limited in part due to its low sensitivity (41%–86%) and poor specificity (33%–100%).^24^, ^25^ Serum CA19‐9 levels may be elevated, even in patients with benign pancreatobiliary disorders such as cholecystitis, cholangitis, and pancreatitis.^26^ In addition, approximately 10% of the entire population does not express Lewis antigens, suggesting that low levels of serum CA 19–9 cannot be used to rule out PC.^27^ Other serologic biomarkers have been suggested as potential screening tools for detecting PC, but none have been proven to be better than that of CA 19–9.^28^

Metabolites are thought to represent the final status of functional responses of the body to environmental stimuli, thereby providing a functional signal derived from the genome‐based proteome that closely reflects the current phenotypic state of an individual. Therefore, the distribution of data regarding serum metabolites in a patient may potentially be used to detect PC.^29^ This may be especially true according to the Warburg effect, in which cancer cells undergo energetically inefficient glycolysis, even in the presence of oxygen‐rich environments (aerobic glycolysis). This may alter the distribution patterns of serum metabolites in patients with PC.^14^

Based on this theory, Yun et al. and Kang et al. attempted to modulate PC‐related metabolites in patients with PC.^30^, ^31^ Meanwhile, Ritchie et al. found for patients with PC that serum metabolites, such as 36‐carbon ultralong‐chain fatty acids, phosphatidylcholines, lysophosphatidylcholines, sphingomyelins, and vinyl ether‐containing plasmalogen ethanolamines, were significantly altered compared with those of healthy controls (all *p* < 0.000025).^13^ Lin et al. and Lacontti et al. investigated the metabolomic changes between pancreatic intraepithelial neoplasia (PanIN) and PC in effort to identify potential serum biomarkers for the early detection of PC in animal models. They demonstrated that some serum metabolites are significantly different between PanIN and PC, suggesting that a more complex set of metabolomic changes occur from noninvasive precursor lesions to invasive cancer.^32^, ^33^

Based on a better understanding of metabolic dysregulation in PC, recent studies have been conducted to diagnose PC with metabolomics. Xie et al. found that a panel of six metabolites (glycerol, glutamine, glycine, proline, serine, and threonine) could discriminate PC from chronic pancreatitis or healthy controls with high accuracy.^34^ This suggests that metabolomics could be useful in differentiating PC from other pancreatic diseases. Moore et al. analyzed a large number of metabolites in plasma samples from patients with different stages of PC and healthy controls. The researchers found that different stages of PC had distinct metabolic profiles, and a combination of metabolites could differentiate pancreatic ductal adenocarcinoma from other types of pancreatic tumors.^35^ Overall, these studies suggest that metabolomics has the potential to aid in the early detection and diagnosis of PC. In the present study, we successfully developed 76‐metabolite‐based diagnostic calculator for the detection of PC and demonstrated fare diagnostic performance for differentiating patients with PC from those with other diseases. In an initial analysis using 116 metabolites (OPLS‐DA, Table S3), the diagnostic performance was found to be excellent in a development cohort, with an accuracy of 0.9886, sensitivity of 0.9825, specificity of 0.9916, and AUC of 0.987. Keeping this high diagnostic performance in mind, we attempted to reduce the number of potential serum metabolites needed to establish diagnostic panels for detecting PC. It was found using 1000 bootstrapping internal validations that at least 76 metabolites were required for the diagnostic panel in order to sustain a high diagnostic performance (Table 4). The difference in AUC values between the OPLS‐DA and SPLS‐DA models was 0.013 (95% CI: −0.016, 0.045), showing no statistical differences in terms of diagnostic performance. The validation cohort showed a lower diagnostic power level compared with that of the development cohort, and although the sensitivity was marked decreased, it still showed high specificity, sensitivity, and accuracy (sensitivity 0.6429, specificity 0.9216, accuracy 0.7822, and AUC 0.7981).

The current study had several strengths. Unlike other previous studies that considered only healthy donors, or chronic pancreatitis as a control^36^, our study included healthy donors and patients with chronic pancreatitis, as well as other benign and malignant conditions, such as gall stones, pancreatic neuroendocrine tumors, thyroid papillary carcinoma, breast cancer, lung cancer, and hepatocellular carcinoma (Table 1). In addition, we believe the present serum 76‐metbolomes diagnostic panel had the highest diagnostic performance reported to date among studies investigating potential diagnostic values of serum metabolites for detecting PC (accuracy 0.977, 95% CI 0.955–0.994; sensitivity 0.964, 95% CI 0.905–1.000; specificity 0.983, 95% CI 0.958–1.000; AUC 0.974, 95% CI 0.944–0.996; Table 4).

No single metabolite has been previously shown to be promising enough for the detection and discrimination of patients with PC. Kobayshi et al. constructed an effective diagnostic model for PC using four serum metabolites, xylitol, 1,5‐anhydro‐D‐glucitol, histidine, and inositol, which were selected from 45 potentially altered metabolites in patients with PC.^36^ Sugimoto, et al. conducted a comprehensive metabolite analysis of saliva samples and identified 57 principal metabolites that can be used in diagnostic models to accurately detect PC, suggesting that cancer‐specific signatures are embedded in salivary metabolites.^37^ Simplifying the diagnostic model by reducing the number of metabolomic signatures detected in patients with PC using high‐diagnostic performance will facilitate the improved clinical feasibility and usefulness of metabolite‐based diagnostic strategies for PC.

Bathe et al. attempted to identify potential serum metabolites to differentiate between benign and malignant pancreatic diseases, and demonstrated a good discrimination power with an AUC of 0.8308.^38^ Leichtle et al. suggested a multivariate model based on specific amino acids in conjunction with CA19‐9, and described a 3‐dimensional analogue of AUC called volume under the ROC surface (VUS) that demonstrated good discrimination (VUS value = 0.89).^39^ Meanwhile, Sugimoto et al. investigated the saliva of unstimulated patients and reported five selected metabolite‐based models with excellent accuracy for the detection of PC with an AUC of 0.94. However, the results from none of the aforementioned studies reach performance levels comparable to those currently observed.^37^

We recently reported the potential role of serum complement factor B in detecting PC.^40^and predicting survival outcomes of patients with resected PC.^41^, ^42^ This new emerging biomarker in conjunction with the current serum 76‐metabolite‐based diagnostic panel may provide the opportunity to improve survival outcomes in the near future of patients with PC. In fact, Mayerle et al. recently identified a biomarker signature of nine metabolites and CA19‐9 for the differential diagnosis between patients with PC and those with chronic pancreatitis, demonstrating that the clinical use of this biomarker signature can improve the diagnosis and treatment stratification of patients compared with that of CA19‐9 alone.^11^ Considering the potential role of neoadjuvant chemotherapy in treating patients with PC, it seems that the long‐term oncologic outcome of PC is less pessimistic, with the hope of cures based on advances in the diagnosis of PC.

The current study had several limitations. For instance, the study had a retrospective design and was based on data from a single ethnicity population with a limited number of study samples. External validation using geographically and demographically different cohorts should be performed. In addition, evaluating 76 metabolites may be too substantial to be practical for clinical oncology. Accordingly, an external validation study using a larger sample size should be performed to in effort to support or improve the diagnostic performance of the current diagnostic panel. Lastly, our study relied on a commercial metabolomics kit. Although other techniques could also quantify these metabolites, the development and validation of robust quantification methods for about hundreds of metabolites will be hard works for individual researchers or clinical laboratory. We selected this commercial kit since it has been analytically validated in the previous study.^40^

In conclusion, we developed a 76‐metabolite‐based diagnostic panel for detecting PC and demonstrated its high diagnostic performance in differentiating patients with PC from those with other diseases.

## AUTHOR CONTRIBUTIONS

**Munseok Choi:** Investigation (equal); methodology (equal); resources (equal); writing – original draft (equal). **Minsu Park:** Funding acquisition (supporting); investigation (equal); methodology (equal); writing – original draft (equal). **Sung Hwan Lee:** Methodology (supporting). **Min Jung Lee:** Methodology (supporting). **Young‐Ki Paik:** Funding acquisition (equal). **Sung Ill Jang:** Resources (supporting). **Dong Ki Lee:** Resources (supporting). **Sang‐Guk Lee:** Conceptualization (equal); investigation (equal); supervision (equal); writing – review and editing (equal). **Chang Moo Kang:** Conceptualization (equal); funding acquisition (equal); supervision (equal); writing – review and editing (equal).

## FUNDING INFORMATION

This research was partly supported by a grant from the Korea Health Technology R&D Project through the Korea Health Industry Development Institute (KHIDI), which was funded by the Ministry of Health & Welfare, Republic of Korea (grant number: HI16C0257), and a National Research Foundation of Korea (NRF) grant funded by the Korean government (MSIT) (grant number: 2021R1C1C1009976 and 2022M3J6A1084843).

## CONFLICT OF INTEREST STATEMENT

The authors have no conflict of interest.

## ETHICS STATEMENT

The study was approved by the Institutional Review Board of Yonsei University College of Medicine (registration date: June 18, 2019; registration number: 4–2019‐0415).

## Supporting information


**Data S1:** SPLS calculator.

**Data S2:** Supplementary figure.

**Data S3:** Supplementary table.

## Data Availability

I confirm that my article contains a Data Availability Statement even if no data is available (list of sample statements) unless my article type does not require one.

## References

[1] Mizrahi JD , Surana R , Valle JW , Shroff RT . Pancreatic cancer. Lancet. 2020;395:2008‐2020. 32593337 10.1016/S0140-6736(20)30974-0

[2] Conlon KC , Klimstra DS , Brennan MF . Long‐term survival after curative resection for pancreatic ductal adenocarcinoma. Clinicopathologic analysis of 5‐year survivors. Ann Surg. 1996;223:273‐279. 8604907 10.1097/00000658-199603000-00007 PMC1235115

[3] Siegel RL , Miller KD , Jemal A . Cancer statistics, 2020. CA Cancer J Clin. 2020;70:7‐30. 31912902 10.3322/caac.21590

[4] Rahib L , Smith BD , Aizenberg R , Rosenzweig AB , Fleshman JM , Matrisian LM . Projecting cancer incidence and deaths to 2030: the unexpected burden of thyroid, liver, and pancreas cancers in the United States. Cancer Res. 2014;74:2913‐2921. 24840647 10.1158/0008-5472.CAN-14-0155

[5] Conroy T , Hammel P , Hebbar M , et al. FOLFIRINOX or gemcitabine as adjuvant therapy for pancreatic cancer. N Engl J Med. 2018;379:2395‐2406. 30575490 10.1056/NEJMoa1809775

[6] Machairas N , Raptis DA , Velázquez PS , et al. The impact of neoadjuvant treatment on survival in patients undergoing pancreatoduodenectomy with concomitant Portomesenteric venous resection: an international multicenter analysis. Ann Surg. 2021;274:721‐728. 34353988 10.1097/SLA.0000000000005132

[7] Smits FJ , Verweij ME , Daamen LA , et al. Impact of complications after pancreatoduodenectomy on mortality, organ failure, hospital stay, and readmission: analysis of a Nationwide audit. Ann Surg. 2022;275:e222‐e228. 32502075 10.1097/SLA.0000000000003835

[8] Kang CM , Lee WJ . Is Laparoscopic Pancreaticoduodenectomy Feasible for Pancreatic Ductal Adenocarcinoma? Cancers (Basel). 2020;12:12. 10.3390/cancers12113430 PMC7699219 33218187

[9] Chu LC , Goggins MG , Fishman EK . Diagnosis and detection of pancreatic cancer. Cancer J. 2017;23:333‐342. 29189329 10.1097/PPO.0000000000000290

[10] Tumas J , Kvederaviciute K , Petrulionis M , et al. Metabolomics in pancreatic cancer biomarkers research. Med Oncol. 2016;33:133. 27807722 10.1007/s12032-016-0853-6

[11] Mayerle J , Kalthoff H , Reszka R , et al. Metabolic biomarker signature to differentiate pancreatic ductal adenocarcinoma from chronic pancreatitis. Gut. 2018;67:128‐137. 28108468 10.1136/gutjnl-2016-312432 PMC5754849

[12] Schmidt DR , Patel R , Kirsch DG , Lewis CA , Vander Heiden MG , Locasale JW . Metabolomics in cancer research and emerging applications in clinical oncology. CA Cancer J Clin. 2021;71:333‐358. 33982817 10.3322/caac.21670 PMC8298088

[13] Ritchie SA , Akita H , Takemasa I , et al. Metabolic system alterations in pancreatic cancer patient serum: potential for early detection. BMC Cancer. 2013;13:416. 24024929 10.1186/1471-2407-13-416 PMC3847543

[14] Urayama S . Pancreatic cancer early detection: expanding higher‐risk group with clinical and metabolomics parameters. World J Gastroenterol. 2015;21:1707‐1717. 25684935 10.3748/wjg.v21.i6.1707 PMC4323446

[15] Rho SY , Lee SG , Park M , et al. Developing a preoperative serum metabolome‐based recurrence‐predicting nomogram for patients with resected pancreatic ductal adenocarcinoma. Sci Rep. 2019;9:18634. 31819109 10.1038/s41598-019-55016-x PMC6901525

[16] Wold H . Estimation of principal components and related models by iterative least squares. Sonderdruck aus. In: Krishnaiah PR , ed. Multivariate Analysis. Academic Press ohne Jahr; 1966:391‐420.

[17] Eriksson L , Trygg J , Wold S . CV‐ANOVA for significance testing of PLS and OPLS® models. J Chemometr. 2008;22:594‐600.

[18] Chung D , Keles S . Sparse partial least squares classification for high dimensional data. Stat Appl Genet Mol Biol. 2010;9:17. 20361856 10.2202/1544-6115.1492 PMC2861314

[19] Vickers AJ , Van Calster B , Steyerberg EW . Net benefit approaches to the evaluation of prediction models, molecular markers, and diagnostic tests. BMJ. 2016;352:i6. 26810254 10.1136/bmj.i6 PMC4724785

[20] Siegel RL , Miller KD , Fuchs HE , Jemal A . Cancer statistics, 2021. CA Cancer J Clin. 2021;71:7‐33. 33433946 10.3322/caac.21654

[21] Jang JY , Kang MJ , Heo JS , et al. A prospective randomized controlled study comparing outcomes of standard resection and extended resection, including dissection of the nerve plexus and various lymph nodes, in patients with pancreatic head cancer. Ann Surg. 2014;259:656‐664. 24368638 10.1097/SLA.0000000000000384

[22] Nickel F , Haney CM , Kowalewski KF , et al. Laparoscopic versus open pancreaticoduodenectomy: a systematic review and meta‐analysis of randomized controlled trials. Ann Surg. 2020;271:54‐66. 30973388 10.1097/SLA.0000000000003309

[23] Philip PA , Lacy J , Portales F , et al. Nab‐paclitaxel plus gemcitabine in patients with locally advanced pancreatic cancer (LAPACT): a multicentre, open‐label phase 2 study. Lancet Gastroenterol Hepatol. 2020;5:285‐294. 31953079 10.1016/S2468-1253(19)30327-9

[24] Herreros‐Villanueva M , Gironella M , Castells A , Bujanda L . Molecular markers in pancreatic cancer diagnosis. Clin Chim Acta. 2013;418:22‐29. 23305796 10.1016/j.cca.2012.12.025

[25] Brand RE , Nolen BM , Zeh HJ , et al. Serum biomarker panels for the detection of pancreatic cancer. Clin Cancer Res. 2011;17:805‐816. 21325298 10.1158/1078-0432.CCR-10-0248 PMC3075824

[26] Bünger S , Laubert T , Roblick UJ , Habermann JK . Serum biomarkers for improved diagnostic of pancreatic cancer: a current overview. J Cancer Res Clin Oncol. 2011;137:375‐389. 21193998 10.1007/s00432-010-0965-x PMC11827947

[27] Parra‐Robert M , Santos VM , Canis SM , Pla XF , Fradera JMA , Porto RM . Relationship between CA 19.9 and the Lewis phenotype: options to improve diagnostic efficiency. Anticancer Res. 2018;38:5883‐5888. 30275214 10.21873/anticanres.12931

[28] Xing H , Wang J , Wang Y , et al. Diagnostic value of CA 19‐9 and carcinoembryonic antigen for pancreatic cancer: a meta‐analysis. Gastroenterol Res Pract. 2018;2018:8704751. 30584422 10.1155/2018/8704751 PMC6280291

[29] Nguyen V , Hurton S , Ayloo S , Molinari M . Advances in pancreatic cancer: the role of metabolomics. Journal of the Pancreas. 2015;16:244‐248.

[30] Kang CM , Yun B , Kim M , et al. Postoperative serum metabolites of patients on a low carbohydrate ketogenic diet after pancreatectomy for pancreatobiliary cancer: a nontargeted metabolomics pilot study. Sci Rep. 2019;9:16820. 31727967 10.1038/s41598-019-53287-y PMC6856065

[31] Yun BK , Song M , Hwang HK , et al. Potential nutritional and metabolomic advantages of high fat Oral supplementation in pancreatectomized pancreaticobiliary cancer patients. Nutrients. 2019;11:893. 31010058 10.3390/nu11040893 PMC6521063

[32] LaConti JJ , Laiakis EC , Mays AD , et al. Distinct serum metabolomics profiles associated with malignant progression in the KrasG12D mouse model of pancreatic ductal adenocarcinoma. BMC Genomics. 2015;16(Suppl 1):S1. 10.1186/1471-2164-16-S1-S1 PMC4315147 25923219

[33] Lin X , Zhan B , Wen S , Li Z , Huang H , Feng J . Metabonomic alterations from pancreatic intraepithelial neoplasia to pancreatic ductal adenocarcinoma facilitate the identification of biomarkers in serum for early diagnosis of pancreatic cancer. Mol Biosyst. 2016;12:2883‐2892. 27400832 10.1039/c6mb00381h

[34] Xie G , Lu L , Qiu Y , et al. Plasma metabolite biomarkers for the detection of pancreatic cancer. J Proteome Res. 2015;14:1195‐1202. 25429707 10.1021/pr501135f PMC4324440

[35] Moore HB , Culp‐Hill R , Reisz JA , et al. The metabolic time line of pancreatic cancer: opportunities to improve early detection of adenocarcinoma. Am J Surg. 2019;218:1206‐1212. 31514959 10.1016/j.amjsurg.2019.08.015

[36] Kobayashi T , Nishiumi S , Ikeda A , et al. A novel serum metabolomics‐based diagnostic approach to pancreatic cancer. Cancer Epidemiol Biomarkers Prev. 2013;22:571‐579. 23542803 10.1158/1055-9965.EPI-12-1033

[37] Sugimoto M , Wong DT , Hirayama A , Soga T , Tomita M . Capillary electrophoresis mass spectrometry‐based saliva metabolomics identified oral, breast and pancreatic cancer‐specific profiles. Metabolomics. 2010;6:78‐95. 20300169 10.1007/s11306-009-0178-y PMC2818837

[38] Bathe OF , Shaykhutdinov R , Kopciuk K , et al. Feasibility of identifying pancreatic cancer based on serum metabolomics. Cancer Epidemiol Biomarkers Prev. 2011;20:140‐147. 21098649 10.1158/1055-9965.EPI-10-0712

[39] Leichtle AB , Ceglarek U , Weinert P , et al. Pancreatic carcinoma, pancreatitis, and healthy controls: metabolite models in a three‐class diagnostic dilemma. Metabolomics. 2013;9:677‐687. 23678345 10.1007/s11306-012-0476-7 PMC3651533

[40] Lee MJ , Na K , Jeong SK , et al. Identification of human complement factor B as a novel biomarker candidate for pancreatic ductal adenocarcinoma. J Proteome Res. 2014;13:4878‐4888. 25057901 10.1021/pr5002719

[41] Hwang HK , Wada K , Kim HY , et al. A nomogram to preoperatively predict 1‐year disease‐specific survival in resected pancreatic cancer following neoadjuvant chemoradiation therapy. Chin J Cancer Res. 2020;32:105‐114. 32194310 10.21147/j.issn.1000-9604.2020.01.12 PMC7072019

[42] Kim SH , Lee MJ , Hwang HK , et al. Prognostic potential of the preoperative plasma complement factor B in resected pancreatic cancer: a pilot study. Cancer Biomark. 2019;24:335‐342. 30829612 10.3233/CBM-181847 PMC13082520

