# Supplementary material for: Development of a metabolite calculator for diagnosis of pancreatic cancer
Source: Cancer Med. 2023 Jun 23;12(15):15933–44. doi: 10.1002/cam4.6233 (PMC10469663; doi:10.1002/cam4.6233)

**Supplementary Figure 1.** A hierarchically clustered heatmap showing differentially expressed metabolites for each category in the development cohort, based on the Euclidean distance calculated from the amount of each metabolite. Yellow and purple represent up- and down standardized values of metabolites, respectively. The metabolites represented in the heatmap are named as in Supplementary Table 3.


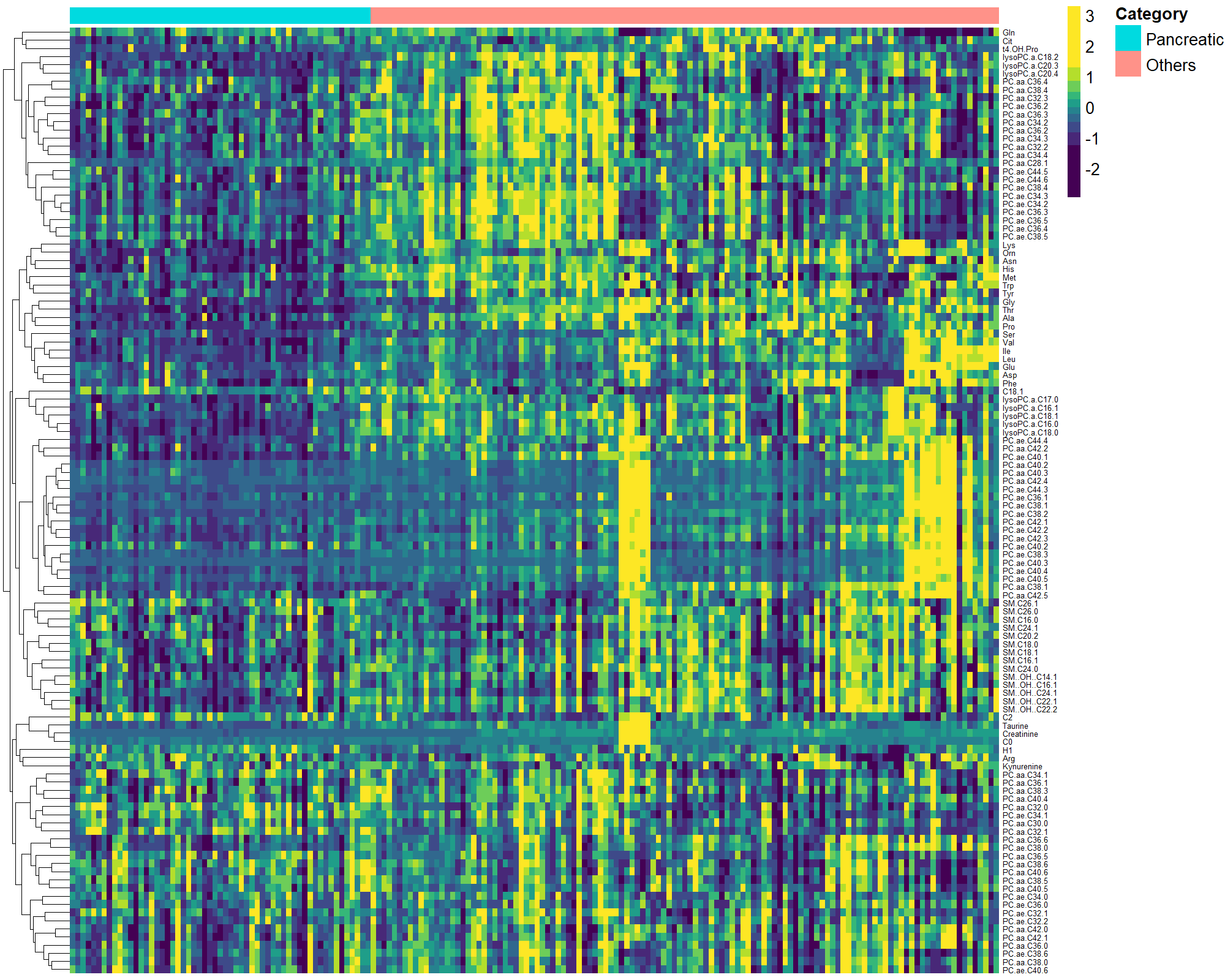

Supplement: Supplementary file 2 — Data S2: Supplementary figure. [file CAM4-12-15933-s001.docx]
