# Supplementary material for: Development of a metabolite calculator for diagnosis of pancreatic cancer
Source: Cancer Med. 2023 Jun 23;12(15):15933–44. doi: 10.1002/cam4.6233 (PMC10469663; doi:10.1002/cam4.6233)
Supplement: Supplementary file 3 — Data S3: Supplementary table. [file CAM4-12-15933-s003.docx]

**Supplementary Table 1.** Spearman’s correlation coefficients between each metabolite and the first OPLS-DA model score

| Metabolite | r^a^ | Metabolite | r^a^ | Metabolite | r^a^ |
| --- | --- | --- | --- | --- | --- |
| Threonine | −0.777 | Hydroxysphingomyeline C22:1 | −0.525 | Hydroxysphingomyeline C24:1 | −0.373 |
| Creatinine | −0.716 | Lysine | −0.511 | Phosphatidylcholine diacyl C36:6 | −0.362 |
| Proline | −0.71 | Phosphatidylcholine acyl-alkyl C36:2 | −0.5 | Phosphatidylcholine acyl-alkyl C42:1 | −0.362 |
| Ornithine | −0.709 | Phosphatidylcholine acyl-alkyl C42:2 | −0.494 | Acetyl-L-carnitine | 0.361 |
| Phosphatidylcholine acyl-alkyl C40:1 | −0.697 | lysoPhosphatidylcholine acyl C18:1 | −0.491 | Phosphatidylcholine diacyl C34:4 | −0.355 |
| Phosphatidylcholine acyl-alkyl C38:2 | −0.669 | Taurine | −0.47 | Phosphatidylcholine diacyl C40:3 | −0.354 |
| Valine | −0.658 | Phosphatidylcholine acyl-alkyl C38:3 | −0.469 | Tryosine | −0.35 |
| Glycine | −0.639 | Phosphatidylcholine acyl-alkyl C40:3 | −0.462 | Phosphatidylcholine acyl-alkyl C40:5 | −0.349 |
| lysoPhosphatidylcholine acyl C18:2 | −0.632 | lysoPhosphatidylcholine acyl C20:3 | −0.461 | Phosphatidylcholine diacyl C42:1 | −0.327 |
| Phosphatidylcholine acyl-alkyl C44:3 | −0.626 | Phosphatidylcholine diacyl C40:2 | −0.455 | Phosphatidylcholine diacyl C32:3 | −0.322 |
| lysoPhosphatidylcholine acyl C16:0 | −0.625 | Hydroxysphingomyeline C22:2 | −0.438 | Phosphatidylcholine acyl-alkyl C36:3 | −0.322 |
| lysoPhosphatidylcholine acyl C18:0 | −0.618 | Phosphatidylcholine diacyl C32:1 | 0.433 | Phosphatidylcholine acyl-alkyl C34:1 | 0.32 |
| lysoPhosphatidylcholine acyl C17:0 | −0.606 | lysoPhosphatidylcholine acyl C16:1 | -−0.43 | Phosphatidylcholine diacyl C42:4 | −0.318 |
| Leucine | −0.601 | trans-4-Hydroxyproline | −0.428 | Octadecenoyl-L-carnitine | 0.312 |
| Phosphatidylcholine acyl-alkyl C38:1 | −0.595 | Phenylalanine | −0.419 | Phosphatidylcholine diacyl C28:1 | −0.312 |
| Phosphatidylcholine acyl-alkyl C42:3 | −0.59 | Phosphatidylcholine diacyl C42:2 | −0.416 | Aspartate | −0.306 |
| Histidine | −0.58 | Phosphatidylcholine diacyl C32:0 | 0.414 | Hydroxysphingomyeline C14:1 | −0.297 |
| Asparagine | −0.569 | Phosphatidylcholine acyl-alkyl C38:0 | −0.414 | Phosphatidylcholine acyl-alkyl C36:4 | −0.287 |
| Phosphatidylcholine acyl-alkyl C44:4 | −0.565 | Citrulline | −0.405 | Phosphatidylcholine acyl-alkyl C44:5 | −0.287 |
| Phosphatidylcholine diacyl C38:1 | −0.56 | Sphingomyeline C24:0 | −0.405 | Phosphatidylcholine diacyl C34:1 | 0.282 |
| Alanine | −0.557 | Sphingomyeline C16:1 | −0.401 | Phosphatidylcholine acyl-alkyl C44:6 | −0.281 |
| Tryptophan | −0.556 | Phosphatidylcholine acyl-alkyl C38:4 | −0.399 | Hydroxysphingomyeline C16:1 | −0.279 |
| Isoleucine | −0.554 | Phosphatidylcholine acyl-alkyl C36:1 | −0.396 | Sphingomyeline C18:1 | −0.276 |
| Serine | −0.553 | Phosphatidylcholine diacyl C32:2 | −0.393 | Phosphatidylcholine acyl-alkyl C34:3 | −0.273 |
| Phosphatidylcholine acyl-alkyl C40:4 | −0.546 | Phosphatidylcholine diacyl C36:2 | −0.383 |  |  |
| lysoPhosphatidylcholine acyl C20:4 | −0.527 | Carnitine | −0.382 |  |  |

OPLS-DA: orthogonal partial least squares discriminant analysis

**Supplementary Table 2.** VIP values derived from the OPLS-DA model.

| Metabolite | VIP | Metabolite | VIP |
| --- | --- | --- | --- |
| Threonine | 2.043 | Alanine | 1.311 |
| Proline | 1.843 | Serine | 1.29 |
| Phosphatidylcholine acyl-alkyl C40:1 | 1.82 | Phosphatidylcholine acyl-alkyl C38:2 | 1.277 |
| Valine | 1.747 | Phosphatidylcholine acyl-alkyl C40:4 | 1.265 |
| Ornithine | 1.743 | Hydroxysphingomyeline C22:2 | 1.25 |
| Histidine | 1.609 | Phosphatidylcholine acyl-alkyl C42:2 | 1.217 |
| lysoPhosphatidylcholine acyl C18:2 | 1.595 | Phosphatidylcholine diacyl C42:2 | 1.178 |
| lysoPhosphatidylcholine acyl C16:0 | 1.578 | lysoPhosphatidylcholine acyl C20:3 | 1.136 |
| lysoPhosphatidylcholine acyl C18:0 | 1.575 | Phosphatidylcholine acyl-alkyl C38:0 | 1.131 |
| Glycine | 1.564 | Phosphatidylcholine diacyl C32:0 | 1.129 |
| Asparagine | 1.515 | Sphingomyeline C24:0 | 1.116 |
| Tryptophan | 1.515 | Citrulline | 1.114 |
| Lysine | 1.475 | Phenylalanine | 1.108 |
| Isoleucine | 1.468 | Phosphatidylcholine diacyl C32:1 | 1.107 |
| Leucine | 1.463 | Phosphatidylcholine acyl-alkyl C44:3 | 1.1 |
| lysoPhosphatidylcholine acyl C17:0 | 1.449 | Phosphatidylcholine acyl-alkyl C38:4 | 1.096 |
| lysoPhosphatidylcholine acyl C20:4 | 1.427 | Phosphatidylcholine diacyl C36:2 | 1.094 |
| Hydroxysphingomyeline C22:1 | 1.413 | Sphingomyeline C16:1 | 1.091 |
| Phosphatidylcholine diacyl C38:1 | 1.408 | Phosphatidylcholine acyl-alkyl C36:1 | 1.065 |
| Phosphatidylcholine acyl-alkyl C42:3 | 1.404 | Phosphatidylcholine diacyl C32:2 | 1.054 |
| Phosphatidylcholine acyl-alkyl C44:4 | 1.379 | Hydroxysphingomyeline C24:1 | 1.023 |
| Phosphatidylcholine acyl-alkyl C36:2 | 1.334 | Phosphatidylcholine acyl-alkyl C38:1 | 1.008 |
| lysoPhosphatidylcholine acyl C18:1 | 1.313 | Tryosine | 1.001 |

VIP: Variable importance in the projection; OPLS-DA: orthogonal partial least squares discriminant analysis

**Supplementary Table 3.** Demographic data of the study subjects

|  | All (n = 176)  Median [Q1, Q3] | Pancreatic Cancer (n = 57)  Median [Q1, Q3] | Others (n = 119)  Median [Q1, Q3] | P-value |
| --- | --- | --- | --- | --- |
| Alanine† | 410740.91 [325230.64, 510123.26] | 458989.90 [380469.12, 533276.88] | 318712.27 [259219.32, 381392.35] | < 0.001 |
| Arginine | 108383.57 [86625.00, 139039.42] | 110190.57 [79531.85, 140560.79] | 108169.81 [97471.70, 128377.36] | 0.706 |
| Asparagine† | 50858.49 [36777.02, 64530.00] | 59567.70 [46103.48, 69777.18] | 36424.84 [28576.40, 42663.35] | < 0.001 |
| Aspartate† | 40358.49 [28420.15, 57435.65] | 42990.57 [32741.99, 61090.55] | 37350.40 [23563.34, 44745.28] | 0.001 |
| Citrulline† | 31587.58 [25648.28, 37228.83] | 33684.67 [28552.79, 39726.64] | 26899.27 [21931.39, 33684.67] | < 0.001 |
| Glutamine | 659179.90 [542245.78, 736006.58] | 662073.43 [498464.90, 744277.51] | 657016.33 [576029.95, 708345.74] | 0.742 |
| Glutamate† | 147712.17 [99035.63, 213581.33] | 157227.72 [111533.42, 231005.63] | 110670.04 [79692.42, 179870.04] | 0.001 |
| Glycine† | 270434.53 [196339.59, 346813.05] | 319073.05 [239671.13, 377511.34] | 194888.29 [158398.57, 228890.08] | < 0.001 |
| Histidine† | 102610.74 [88711.98, 119581.88] | 108793.96 [99436.62, 125536.38] | 85082.61 [70160.87, 93547.83] | < 0.001 |
| Isoleucine† | 90152.73 [74478.63, 115114.70] | 99678.95 [84012.92, 130089.47] | 72951.81 [63959.49, 84350.53] | < 0.001 |
| Leucine† | 185256.43 [151670.58, 239470.11] | 210230.38 [171536.41, 264767.26] | 140489.13 [112391.30, 176250.00] | < 0.001 |
| Lysine† | 288777.30 [237369.36, 334742.41] | 308000.00 [271715.84, 349618.99] | 243449.02 [202874.19, 285422.99] | < 0.001 |
| Methionine | 29159.80 [22790.02, 34757.75] | 31350.98 [23079.41, 37019.61] | 26542.28 [22434.55, 30018.06] | 0.003 |
| Ornithine† | 131284.11 [90211.17, 171480.95] | 156609.93 [124418.58, 201388.75] | 73426.95 [58792.91, 99357.45] | < 0.001 |
| Phenylalanine† | 95834.74 [80869.64, 119739.10] | 104828.08 [85594.18, 131009.65] | 85896.75 [67299.35, 95544.16] | < 0.001 |
| Proline† | 177265.65 [129616.09, 225379.19] | 204744.76 [166269.23, 259842.37] | 119132.44 [103883.49, 140099.75] | < 0.001 |
| Serine† | 198210.95 [160471.95, 276450.70] | 219267.61 [179245.28, 306816.90] | 164772.73 [138636.36, 192045.45] | < 0.001 |
| Threonine† | 105824.95 [71406.51, 160841.78] | 147144.97 [101076.39, 171869.04] | 57628.51 [43598.86, 72350.21] | < 0.001 |
| Tryptophan† | 56190.07 [45386.25, 71257.96] | 65861.25 [51528.75, 77990.66] | 46285.71 [41334.75, 54805.97] | < 0.001 |
| Tyrosine† | 72692.53 [59372.50, 84184.12] | 77375.62 [65402.93, 89552.90] | 64016.75 [55022.66, 73010.84] | < 0.001 |
| Valine† | 271324.82 [234167.97, 321356.19] | 298057.91 [265722.88, 348809.52] | 209379.52 [181051.71, 254950.36] | < 0.001 |
| Creatinine† | 49607.84 [11027.40, 70851.46] | 62809.09 [48588.98, 76439.75] | 10307.28 [9109.97, 12198.68] | < 0.001 |
| Kynurenine | 2058.15 [1748.84, 2419.13] | 2018.65 [1725.54, 2434.41] | 2138.34 [1857.68, 2378.90] | 0.348 |
| trans-4-Hydroxyproline† | 9500.21 [6996.64, 13500.51] | 10903.04 [8179.45, 14814.97] | 6862.12 [5218.18, 9906.06] | < 0.001 |
| Taurine† | 151225.31 [113698.65, 193136.31] | 169188.65 [139994.60, 201539.81] | 106321.47 [73950.38, 145242.72] | < 0.001 |
| Carnitine | 45438.85 [36980.32, 52177.17] | 47993.84 [40811.09, 55013.35] | 39169.67 [32303.10, 46382.38] | < 0.001 |
| Acetyl-L-carnitine† | 7762.73 [5595.32, 9386.40] | 6775.26 [5098.83, 8623.19] | 9085.11 [7709.45, 11924.52] | < 0.001 |
| Octadecenoyl-L-carnitine | 154.72 [122.33, 190.41] | 140.94 [112.10, 184.20] | 171.20 [151.25, 195.36] | < 0.001 |
| lysoPhosphatidylcholine acyl C16:0† | 86183.44 [69201.61, 110114.19] | 94029.03 [82553.24, 124284.99] | 65488.19 [54993.80, 77777.89] | < 0.001 |
| lysoPhosphatidylcholine acyl C16:1† | 2254.14 [1829.71, 2747.75] | 2387.42 [2034.94, 2942.21] | 1861.90 [1521.08, 2174.96] | < 0.001 |
| lysoPhosphatidylcholine acyl C17:0† | 1375.30 [1097.00, 1670.00] | 1540.00 [1310.00, 1889.61] | 1083.22 [856.60, 1308.09] | < 0.001 |
| lysoPhosphatidylcholine acyl C18:0† | 27038.04 [19857.16, 33729.90] | 29974.56 [26012.04, 37567.03] | 18908.82 [15508.85, 22497.59] | < 0.001 |
| lysoPhosphatidylcholine acyl C18:1† | 13512.10 [11892.06, 17778.99] | 15318.81 [12721.63, 19799.33] | 11881.97 [10843.92, 13259.76] | < 0.001 |
| lysoPhosphatidylcholine acyl C18:2† | 17356.79 [12928.32, 26177.12] | 21511.54 [16820.19, 28827.75] | 12387.93 [10744.06, 14370.59] | < 0.001 |
| lysoPhosphatidylcholine acyl C20:3† | 1205.05 [951.72, 1732.40] | 1423.10 [1100.64, 1864.44] | 967.32 [760.93, 1166.63] | < 0.001 |
| lysoPhosphatidylcholine acyl C20:4† | 4361.00 [3358.76, 6001.92] | 4900.22 [4043.17, 6672.83] | 3141.72 [2689.12, 4132.50] | < 0.001 |
| Phosphatidylcholine diacyl C28:1 | 1752.82 [1482.28, 2078.45] | 1878.30 [1529.51, 2222.86] | 1530.96 [1382.36, 1774.11] | < 0.001 |
| Phosphatidylcholine diacyl C30:0 | 2234.02 [1895.22, 2864.90] | 2184.10 [1878.33, 2854.10] | 2407.06 [1946.72, 2864.07] | 0.47 |
| Phosphatidylcholine diacyl C32:0† | 11646.94 [9816.85, 13987.07] | 10701.25 [9339.46, 12602.72] | 13513.64 [11490.34, 15150.00] | < 0.001 |
| Phosphatidylcholine diacyl C32:1† | 9781.06 [7038.83, 13907.28] | 8780.93 [6671.02, 11559.34] | 13170.93 [9046.98, 18722.50] | < 0.001 |
| Phosphatidylcholine diacyl C32:2† | 2215.37 [1652.85, 2937.56] | 2487.77 [1893.29, 3257.62] | 1881.32 [1277.81, 2389.28] | < 0.001 |
| Phosphatidylcholine diacyl C32:3† | 302.03 [241.24, 358.75] | 316.01 [260.00, 388.61] | 267.95 [229.34, 308.11] | < 0.001 |
| Phosphatidylcholine diacyl C34:1 | 158939.74 [137253.00, 195285.83] | 152721.14 [130791.83, 188456.03] | 175682.74 [151615.11, 208425.30] | 0.003 |
| Phosphatidylcholine diacyl C34:2† | 277449.74 [232366.38, 333862.07] | 298148.60 [237569.78, 359650.19] | 248364.82 [216199.63, 282258.57] | < 0.001 |
| Phosphatidylcholine diacyl C34:3 | 10776.62 [8758.50, 13195.52] | 11189.86 [9445.00, 13774.14] | 10214.35 [7867.48, 12317.92] | 0.021 |
| Phosphatidylcholine diacyl C34:4† | 845.19 [620.40, 1114.98] | 932.03 [684.92, 1215.37] | 708.68 [557.58, 888.15] | < 0.001 |
| Phosphatidylcholine diacyl C36:0 | 2499.85 [2099.68, 3209.83] | 2652.86 [2185.34, 3272.50] | 2335.25 [1854.66, 2867.66] | 0.008 |
| Phosphatidylcholine diacyl C36:1 | 36073.15 [28689.77, 44091.49] | 36316.91 [29955.25, 45542.35] | 35702.73 [27249.04, 39559.68] | 0.044 |
| Phosphatidylcholine diacyl C36:2† | 169923.70 [128755.68, 201641.15] | 186244.31 [150500.11, 225277.46] | 142076.46 [114168.60, 168824.66] | < 0.001 |
| Phosphatidylcholine diacyl C36:3† | 84228.78 [68707.27, 102405.90] | 86896.93 [71616.19, 105724.72] | 76359.28 [62902.62, 86917.11] | 0.001 |
| Phosphatidylcholine diacyl C36:4 | 129216.82 [107893.02, 152636.87] | 132032.93 [107533.18, 158895.88] | 122638.06 [108811.72, 139302.01] | 0.153 |
| Phosphatidylcholine diacyl C36:5 | 25881.01 [19059.78, 36404.47] | 24160.95 [18372.26, 34437.56] | 29976.13 [23888.62, 39683.70] | 0.006 |
| Phosphatidylcholine diacyl C36:6† | 950.42 [720.05, 1246.83] | 991.99 [790.01, 1410.88] | 793.95 [606.91, 1026.79] | < 0.001 |
| Phosphatidylcholine diacyl C38:0 | 3184.00 [2635.63, 3948.71] | 3304.45 [2734.77, 4025.00] | 2901.88 [2603.41, 3679.40] | 0.041 |
| Phosphatidylcholine diacyl C38:1† | 963.82 [632.45, 1582.57] | 1313.80 [830.20, 1937.32] | 641.81 [499.88, 812.59] | < 0.001 |
| Phosphatidylcholine diacyl C38:3 | 33002.93 [27929.06, 41208.25] | 33717.33 [28559.78, 41656.89] | 32341.00 [26497.99, 40493.27] | 0.457 |
| Phosphatidylcholine diacyl C38:4 | 76529.14 [63127.61, 89824.69] | 80681.99 [65143.10, 93957.27] | 70372.14 [59663.89, 82965.53] | 0.005 |
| Phosphatidylcholine diacyl C38:5 | 45008.39 [37418.46, 56302.34] | 43842.86 [35345.07, 56479.05] | 47569.85 [39859.21, 55875.41] | 0.086 |
| Phosphatidylcholine diacyl C38:6 | 104725.83 [82051.51, 133380.31] | 96694.11 [76404.79, 127880.76] | 116877.91 [96614.97, 139910.75] | 0.002 |
| Phosphatidylcholine diacyl C40:2† | 261.50 [208.17, 334.58] | 293.88 [234.40, 380.41] | 209.12 [168.06, 261.64] | < 0.001 |
| Phosphatidylcholine diacyl C40:3† | 437.03 [381.01, 536.29] | 466.57 [401.27, 585.29] | 390.46 [344.99, 454.71] | < 0.001 |
| Phosphatidylcholine diacyl C40:4 | 2305.50 [1922.85, 2798.25] | 2356.68 [2014.95, 2937.80] | 2248.93 [1750.28, 2613.52] | 0.071 |
| Phosphatidylcholine diacyl C40:5 | 8719.01 [6704.92, 10541.42] | 8413.57 [6736.32, 10629.55] | 8897.73 [6602.34, 10310.02] | 0.923 |
| Phosphatidylcholine diacyl C40:6 | 36369.55 [27622.78, 46470.52] | 34561.71 [26373.24, 45542.82] | 39774.66 [30958.27, 49620.68] | 0.079 |
| Phosphatidylcholine diacyl C42:0 | 561.41 [483.68, 708.13] | 610.12 [504.20, 726.60] | 535.31 [445.28, 619.26] | 0.008 |
| Phosphatidylcholine diacyl C42:1† | 261.04 [224.63, 337.34] | 290.11 [235.48, 345.57] | 232.49 [194.20, 273.52] | < 0.001 |
| Phosphatidylcholine diacyl C42:2† | 288.48 [230.46, 373.07] | 309.52 [253.55, 415.76] | 241.08 [194.09, 294.19] | < 0.001 |
| Phosphatidylcholine diacyl C42:4† | 163.68 [137.77, 198.98] | 175.52 [143.79, 220.05] | 151.74 [115.23, 167.72] | < 0.001 |
| Phosphatidylcholine diacyl C42:5† | 319.13 [250.22, 427.30] | 327.78 [261.52, 464.17] | 284.60 [237.67, 370.49] | 0.006 |
| Phosphatidylcholine acyl-alkyl C32:1 | 2145.96 [1806.49, 2479.30] | 2105.72 [1805.22, 2500.16] | 2169.48 [1809.81, 2474.39] | 0.869 |
| Phosphatidylcholine acyl-alkyl C32:2† | 597.10 [521.59, 715.46] | 631.80 [525.04, 760.13] | 554.35 [514.40, 621.20] | 0.003 |
| Phosphatidylcholine acyl-alkyl C34:0 | 1009.66 [822.76, 1228.71] | 999.14 [809.83, 1226.58] | 1049.75 [856.33, 1233.80] | 0.498 |
| Phosphatidylcholine acyl-alkyl C34:1 | 6054.98 [5246.75, 7042.15] | 5731.47 [5045.25, 6742.82] | 6559.37 [5783.79, 7541.01] | 0.001 |
| Phosphatidylcholine acyl-alkyl C34:2† | 7143.24 [5505.04, 9797.92] | 7786.84 [5565.93, 10505.03] | 6299.73 [5439.67, 7235.40] | < 0.001 |
| Phosphatidylcholine acyl-alkyl C34:3† | 5395.16 [3977.39, 7331.07] | 5876.90 [4025.63, 8264.50] | 4612.54 [3820.18, 5616.89] | < 0.001 |
| Phosphatidylcholine acyl-alkyl C36:0 | 937.90 [763.10, 1169.41] | 960.23 [754.39, 1196.90] | 900.60 [769.06, 1042.66] | 0.19 |
| Phosphatidylcholine acyl-alkyl C36:1† | 5072.22 [4379.91, 6226.12] | 5488.10 [4570.14, 6603.78] | 4492.28 [3936.74, 5116.83] | < 0.001 |
| Phosphatidylcholine acyl-alkyl C36:2† | 8755.61 [6916.91, 10311.65] | 9289.07 [8307.03, 11264.97] | 6955.42 [5973.36, 8025.48] | < 0.001 |
| Phosphatidylcholine acyl-alkyl C36:3† | 4732.51 [3639.26, 5774.57] | 5262.14 [3849.71, 6883.47] | 4016.18 [3423.01, 4670.13] | < 0.001 |
| Phosphatidylcholine acyl-alkyl C36:4† | 11664.02 [9276.53, 14051.06] | 12529.91 [9534.95, 17025.89] | 10501.39 [8531.91, 12106.08] | < 0.001 |
| Phosphatidylcholine acyl-alkyl C36:5 | 8923.36 [7142.11, 10710.46] | 9295.28 [7180.50, 11209.46] | 8754.21 [7117.54, 9999.73] | 0.108 |
| Phosphatidylcholine acyl-alkyl C38:0† | 2054.12 [1713.04, 2747.98] | 2211.66 [1836.16, 2998.51] | 1813.19 [1557.72, 2150.00] | < 0.001 |
| Phosphatidylcholine acyl-alkyl C38:1† | 498.06 [337.97, 839.01] | 692.30 [449.55, 1102.59] | 320.81 [218.24, 404.87] | < 0.001 |
| Phosphatidylcholine acyl-alkyl C38:2† | 1504.07 [1104.68, 1983.38] | 1741.50 [1445.88, 2230.62] | 1005.56 [887.04, 1225.00] | < 0.001 |
| Phosphatidylcholine acyl-alkyl C38:3† | 2725.67 [2379.35, 3371.55] | 3050.00 [2592.21, 3650.00] | 2382.53 [2142.54, 2680.73] | < 0.001 |
| Phosphatidylcholine acyl-alkyl C38:4† | 7619.36 [6596.96, 9446.98] | 8541.18 [7015.66, 9959.36] | 6854.40 [5665.82, 7922.47] | < 0.001 |
| Phosphatidylcholine acyl-alkyl C38:5 | 11487.97 [9356.30, 13510.46] | 11753.13 [9386.87, 14356.13] | 11132.60 [9368.17, 12657.79] | 0.083 |
| Phosphatidylcholine acyl-alkyl C38:6 | 6932.65 [5463.35, 8300.57] | 6948.69 [5437.03, 8401.49] | 6442.65 [5497.04, 8053.80] | 0.492 |
| Phosphatidylcholine acyl-alkyl C40:1† | 1020.44 [769.02, 1365.00] | 1229.33 [999.18, 1544.33] | 675.75 [581.23, 849.72] | < 0.001 |
| Phosphatidylcholine acyl-alkyl C40:2 | 1364.38 [1151.45, 1687.71] | 1370.25 [1151.04, 1762.95] | 1352.83 [1156.33, 1656.50] | 0.476 |
| Phosphatidylcholine acyl-alkyl C40:3† | 784.58 [643.10, 978.93] | 869.98 [727.64, 1132.26] | 650.17 [593.46, 740.30] | < 0.001 |
| Phosphatidylcholine acyl-alkyl C40:4† | 1704.02 [1387.92, 2081.21] | 1849.47 [1621.96, 2425.46] | 1301.52 [1090.82, 1576.56] | < 0.001 |
| Phosphatidylcholine acyl-alkyl C40:5† | 2931.36 [2399.55, 3523.51] | 3116.27 [2588.13, 3959.35] | 2472.59 [2163.78, 3071.10] | < 0.001 |
| Phosphatidylcholine acyl-alkyl C40:6 | 4277.14 [3347.32, 5147.29] | 4383.00 [3357.79, 5088.57] | 4102.40 [3259.99, 5147.73] | 0.601 |
| Phosphatidylcholine acyl-alkyl C42:1† | 295.69 [245.82, 365.24] | 309.09 [266.61, 388.73] | 258.33 [227.26, 309.82] | < 0.001 |
| Phosphatidylcholine acyl-alkyl C42:2† | 489.51 [392.92, 617.06] | 534.81 [422.94, 705.06] | 416.93 [332.89, 486.78] | < 0.001 |
| Phosphatidylcholine acyl-alkyl C42:3† | 681.64 [552.58, 833.06] | 747.55 [647.06, 951.13] | 538.86 [435.01, 629.00] | < 0.001 |
| Phosphatidylcholine acyl-alkyl C44:3† | 92.82 [73.61, 132.97] | 113.66 [88.37, 156.30] | 70.79 [60.26, 84.95] | < 0.001 |
| Phosphatidylcholine acyl-alkyl C44:4† | 246.67 [213.33, 297.09] | 267.67 [234.12, 331.40] | 214.29 [184.63, 231.51] | < 0.001 |
| Phosphatidylcholine acyl-alkyl C44:5† | 987.24 [820.10, 1158.05] | 1048.95 [844.18, 1215.37] | 851.98 [778.47, 999.01] | < 0.001 |
| Phosphatidylcholine acyl-alkyl C44:6† | 1049.49 [861.45, 1258.41] | 1089.02 [944.84, 1310.28] | 932.15 [811.58, 1138.29] | < 0.001 |
| Hydroxysphingomyeline C14:1 | 3713.33 [2998.56, 4326.99] | 3865.14 [3098.87, 4469.79] | 3391.41 [2865.76, 4006.61] | 0.008 |
| Hydroxysphingomyeline C16:1 | 2431.20 [1986.89, 2785.37] | 2516.25 [2018.38, 2832.46] | 2211.89 [1960.48, 2575.63] | 0.016 |
| Hydroxysphingomyeline C22:1† | 8523.00 [6738.53, 10190.18] | 9355.49 [7797.05, 11024.47] | 6723.87 [5645.28, 7999.70] | < 0.001 |
| Hydroxysphingomyeline C22:2† | 7239.58 [5898.87, 8804.08] | 7888.59 [6087.74, 9791.94] | 6501.24 [5175.39, 7376.37] | < 0.001 |
| Hydroxysphingomyeline C24:1† | 899.83 [743.05, 1104.29] | 947.07 [776.95, 1208.32] | 823.75 [701.21, 937.78] | < 0.001 |
| Sphingomyeline C16:0 | 98082.12 [85359.91, 110657.22] | 100616.69 [86575.52, 113902.67] | 93299.06 [81112.49, 102038.63] | 0.01 |
| Sphingomyeline C16:1† | 12659.81 [10782.42, 14671.68] | 13273.20 [11618.52, 15396.91] | 11382.86 [9387.25, 13062.03] | < 0.001 |
| Sphingomyeline C18:0 | 19247.20 [16648.37, 23098.04] | 19900.50 [16965.67, 24565.62] | 18400.39 [15560.32, 21731.59] | 0.055 |
| Sphingomyeline C18:1† | 9401.36 [7946.83, 11189.52] | 9671.69 [8188.05, 11661.96] | 8799.67 [7468.50, 10148.23] | 0.005 |
| Sphingomyeline C20:2 | 297.80 [236.02, 365.43] | 314.98 [240.00, 383.88] | 261.84 [215.71, 315.45] | 0.007 |
| Sphingomyeline C24:0† | 16227.73 [13675.68, 18967.19] | 17281.08 [15206.33, 19674.14] | 13868.78 [11984.98, 16626.64] | < 0.001 |
| Sphingomyeline C24:1 | 45397.80 [38446.02, 54611.36] | 45583.18 [38406.59, 55993.18] | 45278.70 [38668.59, 52192.96] | 0.512 |
| Sphingomyeline C26:0 | 124.74 [98.89, 158.25] | 127.48 [99.23, 159.56] | 124.19 [93.06, 142.35] | 0.335 |
| Sphingomyeline C26:1† | 277.23 [224.93, 343.36] | 260.82 [208.86, 321.27] | 310.91 [266.00, 367.91] | < 0.001 |
| Hexose | 5053326.27 [4327455.31, 5912622.80] | 4819868.05 [4239358.96, 5613840.92] | 5639412.02 [4701146.99, 6257875.03] | 0.002 |

Continuous variables are presented as median values with interquartile range (IQR). The subscript “†” indicates the selected metabolites through SPLS-DA.

**Supplementary Table 4. Seventy-two metabolites excluded from statistical analysis.**

| Metabolite | Full name | % (below LOD + no detection) in our study | % (below LOD + no detection) in the previous study* |
| --- | --- | --- | --- |
| Ac-Orn | Acetylornithine | 3.3 | 42.8 |
| ADMA | Asymmetric dimethylarginine | 74.3 | 2 |
| alpha-AAA | alpha-Aminoadipic acid | 30.3 | 16.4 |
| c4-OH-Pro | cis-4-Hydroxyproline | 97.9 | 83.9 |
| Carnosine | Carnosine | 97.1 | 92.2 |
| DOPA | DOPA | 97.5 | 92.2 |
| Dopamine | Dopamine | 91.7 | 100 |
| Histamine | Histamine | 56.4 | 82.8 |
| Met-SO | Methioninesulfoxide | 18.3 | 34.2 |
| Nitro-Tyr | Nitrotyrosine | 95.4 | 98.3 |
| PEA | Phenylethylamine | 98.8 | 95.6 |
| Putrescine | Putrescine | 19.1 | 35.6 |
| SDMA | Symmetric dimethylarginine | 51.0 | 10.8 |
| Serotonin | Serotonin | 2.1 | 3.1 |
| Spermidine | Spermidine | 7.1 | 14.2 |
| Spermine | Spermine | 12.4 | 35.3 |
| total DMA | Total dimethylarginine | 2.9 | 33.3 |
| C10 | Decanoyl-L-carnitine | 90.0 | 9.7 |
| C10:1 | Decenoyl-L-carnitine | 78.0 | 68.6 |
| C10:2 | Decadienyl-L-carnitine | 73.0 | 74.7 |
| C12 | Dodecanoyl-L-carnitine | 75.9 | 13.9 |
| C12:1 | Dodecenoyl-L-carnitine | 98.8 | 70 |
| C12-DC | Dodecanedioyl-L-carnitine | 100.0 | 100 |
| C14 | Tetradecanoyl-L-carnitine | 71.8 | 50.6 |
| C14:1 | Tetradecenoyl-L-carnitine | 46.9 | 2.8 |
| C14:1-OH | Hydroxytetradecenoyl-L-carnitine | 90.5 | 53.3 |
| C14:2 | Tetradecadienyl-L-carnitine | 65.1 | 18.3 |
| C14:2-OH | Hydroxytetradecadienyl-L-carnitine | 83.4 | 78.3 |
| C16 | Hexadecanoyl-L-carnitine | 5.0 | 0.6 |
| C16:1 | Hexadecenoyl-L-carnitine | 88.4 | 67.2 |
| C16:1-OH | Hydroxyhexadecenoyl-L-carnitine | 100.0 | 83.1 |
| C16:2 | Hexadecadienyl-L-carnitine | 72.6 | 75.6 |
| C16:2-OH | Hydroxyhexadecadienyl-L-carnitine | 99.6 | 92.5 |
| C16-OH | Hydroxyhexadecanoyl-L-carnitine | 72.6 | 83.1 |
| C18 | Octadecanoyl-L-carnitine | 36.1 | 7.2 |
| C18:1-OH | Hydroxyoctadecenoyl-L-carnitine | 96.7 | 85.6 |
| C18:2 | Octadecadienyl-L-carnitine | 3.3 | 6.7 |
| C3 | Propionyl-L-carnitine | 9.5 | 0.6 |
| C3:1 | Propenyl-L-carnitine | 100.0 | 76.1 |
| C3-DC (C4-OH) | Malonyl-L-carnitine/Hydroxybutyryl-L-carnitine | 97.5 | 29.4 |
| C3-OH | Hydroxypropionyl-L-carnitine | 100.0 | 72 |
| C4 | Butyryl-L-carnitine | 36.9 | 0.6 |
| C4:1 | Butenyl-L-carnitine | 98.3 | 70.6 |
| C5 | Valeryl-L-carnitine | 48.1 | 1.9 |
| C5:1 | Tiglyl-L-carnitine | 88.8 | 82.8 |
| C5:1-DC | Glutaconyl-L-carnitine | 73.0 | 73.6 |
| C5-DC (C6-OH) | Glutaryl-L-carnitine/Hydroxyhexanoyl-L-carnitine | 99.6 | 65 |
| C5-M-DC | Methylglutaryl-L-carnitine | 99.2 | 100 |
| C5-OH (C3-DC-M) | Methylmalonyl-L-carnitine/Hydroxyvaleryl-L-carnitine | 100.0 | 61.7 |
| C6 (C4:1-DC) | Fumaryl-L-carnitine/Hexanoyl-L-carnitine | 94.2 | 38.3 |
| C6:1 | Hexenoyl-L-carnitine | 100.0 | 82 |
| C7-DC | Pimelyl-L-carnitine | 97.5 | 41.1 |
| C8 | Octanoyl-L-carnitine | 99.2 | 30.3 |
| C9 | Nonayl-L-carnitine | 97.9 | 41.4 |
| lysoPC a C14:0 | lysoPhosphatidylcholine acyl C14:0 | 90.0 | 95 |
| lysoPC a C24:0 | lysoPhosphatidylcholine acyl C24:0 | 63.1 | 22.5 |
| lysoPC a C26:0 | lysoPhosphatidylcholine acyl C26:0 | 87.1 | 3.9 |
| lysoPC a C26:1 | lysoPhosphatidylcholine acyl C26:1 | 65.6 | 2 |
| lysoPC a C28:0 | lysoPhosphatidylcholine acyl C28:0 | 61.4 | 17.2 |
| lysoPC a C28:1 | lysoPhosphatidylcholine acyl C28:1 | 12.0 | 0.6 |
| PC aa C24:0 | Phosphatidylcholine diacyl C24:0 | 71.4 | 27 |
| PC aa C26:0 | Phosphatidylcholine diacyl C26:0 | 100.0 | 80.8 |
| PC aa C30:2 | Phosphatidylcholine diacyl C30:2 | 29.0 | 34.2 |
| PC aa C40:1 | Phosphatidylcholine diacyl C40:1 | 28.2 | 45.8 |
| PC aa C42:6 | Phosphatidylcholine diacyl C42:6 | 7.1 | 14.2 |
| PC ae C30:0 | Phosphatidylcholine acyl-alkyl C30:0 | 6.6 | 0.6 |
| PC ae C30:1 | Phosphatidylcholine acyl-alkyl C30:1 | 12.9 | 16.4 |
| PC ae C30:2 | Phosphatidylcholine acyl-alkyl C30:2 | 41.1 | 0.6 |
| PC ae C42:0 | Phosphatidylcholine acyl-alkyl C42:0 | 71.4 | 90 |
| PC ae C42:4 | Phosphatidylcholine acyl-alkyl C42:4 | 4.1 | 0.6 |
| PC ae C42:5 | Phosphatidylcholine acyl-alkyl C42:5 | 5.0 | 0.6 |
| SM C22:3 | Sphingomyeline C22:3 | 65.6 | 72.2 |

**Supplementary Table 5. LC parameters used for metabolites measurement**

|  | Time (min) | Flow rate (mL/min) | Mobile phase A (%) |
| --- | --- | --- | --- |
| LC-MS/MS | 0.0 | 0.5 | 100 |
|  | 0.5 | 0.5 | 100 |
|  | 5.5 | 0.5 | 5 |
|  | 6.5 | 0.5 | 5 |
|  | 7.0 | 0.5 | 100 |
|  | 9.5 | 0.5 | 100 |
| FIA-MS/MS | 0.0 | 0.03 | MS mobile phase prepared by Biocrates Solvent Ⅰ in isocratic mode |
|  | 1.6 | 0.03 |  |
|  | 2.4 | 0.2 |  |
|  | 2.8 | 0.2 |  |
|  | 3.0 | 0.03 |  |

LC-MS/MS, liquid chromatography-tandem mass spectrometry; FIA, flow injection analysis.

**Supplementary Table 6. MS parameters used for metabolites measurement**

| Instrument | Parameter | LC‐MS/MS | FIA-MS/MS | |
| --- | --- | --- | --- | --- |
|  |  |  | Positive mode | Negative mode |
| QTRAP 5500 | CUR (psi) | 20 | 20 | 20 |
|  | IS (V) | 5500 | 5500 | -4500 |
|  | TEM (°C) | 500 | 200 | 200 |
|  | GS1 (psi) | 40 | 40 | 40 |
|  | GS2 (psi) | 50 | 50 | 50 |
|  | CAD (psi) | 6 | 6 | 6 |
|  | EP (V) | 10 | 10 | 10 |

CUR, curtain gas; IS, ion spray voltage; TEM, temperature; GS1/GS2, ion source gas 1 and 2; CAD, collisionally activated dissociation gas; EP, entrance potential; LC-MS/MS, liquid chromatography-tandem mass spectrometry; FIA, flow injection analysis.
